# Secure and reliable blockchain-based eBook transaction system for self-published eBook trading

**DOI:** 10.1371/journal.pone.0228418

**Published:** 2020-02-03

**Authors:** Jeonghee Chi, Jangyeon Lee, Nakyung Kim, Jeewoo Choi, Soyoung Park

**Affiliations:** Department of Software, Konkuk University, Seoul, South Korea; Wuhan University, CHINA

## Abstract

As eBook readers have expanded on the market, various online eBook markets have arisen as well. Currently, the online eBook market consists of at least publishers and online platform providers and authors, and these actors inevitably incur intermediate costs between them. In this paper, we introduce a blockchain-based eBook market system that enables self-published eBook trading and direct payments from readers to authors without any trusted party; because authors publish themselves and readers purchase directly from authors, neither actor incurs any intermediate costs. However, because of this trustless environment, the validity, ownership and intellectual property of digital contents cannot be verified and protected, and the safety of purchase transactions cannot be ensured. To address these shortcomings, we propose a secure and reliable eBook transaction system that satisfies the following security requirements: (1) verification of the ownership of each eBook, (2) confidentiality of eBook contents, (3) authorization of a right to read a book, (4) authentication of a legitimate purchaser, (5) verification of the validity and integrity of eBook contents, (6) safety of direct purchase transactions, and (7) preventing eBook piracy and illegal distribution. We provide practical cryptographic protocols for the proposed system and analyze the security and simulated performance of the proposed schemes.

## Introduction

An eBook, also known as an electronic or digital book, is a digitally released version of a book, often consisting of text and images and available on electronic devices. According to statista.com [1], e-books have become popular among American book readers– 20 percent of book readers in the United States stated that they read more eBooks than hard copy books, and 23 percent read about the same number of hard copy books and eBooks. In South Korea, many eBook specialized platforms including ridibooks [2], Millie [3], and Series [4] have recently launched. Demand for eBooks has been increased steadily, and the number of self-published eBooks has risen dramatically in the last decade. In 2017, there were a total of over one million print and eBooks self-published in the United States, of which 879,600 were print books and 129,600 were eBooks [5]. Currently, the eBook market system consists in essence of publishers, online platform providers (or shops), and authors; the online eBook platform serves as a trusted party for secure distribution and management of digital contents. The platform contracts with the author or publisher and hosts the sales of the digital contents; then the sales revenue is distributed between the two. Therefore, an intermediate fee occurs for the publisher and distributor.

With the advent of YouTube, publishing, sharing and subscribing to personal creations has become very popular today. Even for eBook contents, a platform that anyone can freely publish, share and sell personal eBook contents is necessary. Many web-novels in Korea, mostly serialized fiction, are published regularly two or three times a week. The first few series can be previewed for free, and after the story raises its awareness, the next series will have to be paid for subscription. An open eBook trading platform will allow more people to freely share and sell their personal novels. The goal of our research is to develop a real-time eBook trading platform over a peer-to-peer (P2P) network where anyone can publish their own eBook contents and buy a right to read the contents from authors. Because the authors publish themselves and receive direct payments from readers, authors incur no royalty costs for publishers, authors only pay a minimum fee for using the eBook platform, and readers benefit from lower book prices. Here, the most difficult issue is that the proposed model allows the sale of personal eBook contents rather than just sharing them, so the proposed model should be able to ensure the reliability of eBook contents, copyright protection and the safety of eBook purchase transactions. With emerging of the blockchain technology [6–15], the blockchain-based smart contract [16], data sharing [17], and DRM technology [18] have been proposed. The blockchain, a distributed database that maintains a continuously increasing list of ordered records, is trusted for correctness and availability because all data recorded in the blockchain is publicly validated.

In this paper, we introduce a secure and reliable blockchain-based eBook market system that enables self-published eBook trading and direct payments from readers to authors without outside trusted parties; users can be either authors or readers. However, owing to the trustless environment, no one can take responsibility not only for the validity, ownership, and intellectual property of digital contents but also for the safety of purchase transactions. Our proposed eBook market system satisfies the following security requirements: (1) verification of the ownership of each eBook, (2) confidentiality of eBook contents, (3) authorization of a right to read a book, (4) authentication of a legitimate purchaser, (5) verification of the validity and integrity of eBook contents, (6) safety of direct purchase transactions, and (7) prevention of eBook piracy and illegal distribution.

The key strategies to achieve our goal are that (1) every eBook information containing the author information is digitally signed with the author’s private key, deployed over the network, and recorded on the blockchain named *B_Chain*; (2) every eBook content is encrypted with its own book key; (3) an eBook’s content is divided into many small book pieces and stored in a book repository as encrypted; (4) only a legitimate purchaser can obtain the book key and the set of encrypted pieces of the purchased book; (5) a buyer can restore a part of the content of a randomly selected book piece during the purchasing process and verify the validity and integrity of the content; (6) every eBook purchase transaction is signed by both the buyer and the author, deployed over the network, and recorded on the blockchain named *C_Chain*, and (7) every purchased eBook’s content is re-encrypted and stored with a new book key so that only the buyer’s service application can restore the content.

Since every eBook information is recorded on *B_Chain* and shared among users in a distributed way, the ownership and copyright of the book can be publicly proved. Buyers and authors directly conduct the eBook purchase transactions, during which the buyer can validate the book’s content and the book key for access; the buyer requests a randomly selected piece of book and decrypts it with the received book key to confirm the content. In addition, the system compares the hash value of the restored book piece with the registered hash value, thereby validating both the book key and the eBook content during the purchasing process. The proposed challenge-and-response protocol combines both advantages of hash-based message authentication and provable data possession (PDP) that generates proofs of possession for randomly selected sample data blocks. The system reveals only a randomly selected piece of book not the entire eBook content. With this revealing process, comparing the hash values is enough for the content verification, a complex homomorphic verifiable cryptographic technique used in the PDP is unnecessary. In addition, the buyer can verify if the revealed book texts make sense or not. It is very effective and suitable for real-time content verification.

When the buyer and the author complete the purchase transaction by mutual agreement, *C_Chain* for the transaction is updated, and deployed over the network so that no purchase transactions can be either forged or repudiated. The buyer with a valid purchase transaction recorded on *C_Chain* can download all the book pieces of the purchased eBook from the book repository. That is, if a buyer acquires only a book key during the book purchase process but does not complete the purchase process, a valid blockchain cannot be generated, and it is impossible for the buyer to obtain the eBook content from the book repository; that is, the book key alone is useless, which in turn protects the eBook content from a malicious buyer.

Traditional copyright protection technologies focus on detecting the data owner or tracking illegal distributers using with watermarking and fingerprinting techniques. In contrast, the proposed model protects the illegal distribution of copyrighted contents by preventing even legitimate buyers from accessing to the original content. All downloaded eBook contents are newly encrypted by the buyer’s service application and stored in the buyer’s local storage. In other words, buyers of the same eBook content receive differently encrypted copies of the book, and the book key they obtained during the eBook purchase is subsequently useless. Because the original plain content of the purchased eBook is neither exposed nor stored anywhere, it is impossible for even the legal purchaser to redistribute or resale the original content to others. Even if an encrypted version stored in the buyer’s local storage is distributed to others, the other service applications cannot reveal the encrypted content because the service applications cannot produce a valid book key for the content.

Consequently, this paper provides a new real-time eBook trading platform that supports self-publishing and direct payments between users on a P2P network without trusted parties. The proposed model uses blockchain technology to effectively protect copyrights on paid contents, and securely manage direct payment transactions.

- All eBook information is publicly verified with *B_Chain*.
- All eBook purchase transactions are publicly verified with *C_Chain*.
- Only validated eBooks registered in *B_Chain* are uploaded to the book repository. Only legitimate buyers can download eBooks from the book repository with their purchase transactions recorded in *C_Chain*. Thus, only verifiable eBooks are shared among users on the network and only legitimate buyers can obtain encrypted pieces of books for their purchased books.

The proposed model suggests a practical purchasing protocol with content verification suitable for real-time services.

- The purchase process includes not only making a payment for the purchased book, but also securing the right to read the book. Therefore, validating the book key provided by the author and verifying the encrypted content stored in the book repository is essential to the buyer. The proposed hash-based challenge-and-response protocol effectively resolves this issue without restoring the entire encrypted content before the purchase is completed.
- The purchasing process is interactive between the buyer and the author and requires signatures from both parties. Hence, a valid purchase transaction can only be created by mutual agreement between the author and buyer.
- The proposed model effectively prevents piracy and illegal distribution on paid content.
- All purchased eBook contents are stored re-encrypted in the buyer’s local device. Access to the original content of the purchased eBook is unavailable, which prevents from being copied or distributed.

The rest of this paper proceeds as follows. In Section 2 and Section 3, we review related works and briefly describe the elliptic curve cryptography [19–21] closely associated with the security of our system. In Section 4, we provide concrete cryptographic protocols that satisfy all the requirements mentioned above. We analyze the security and efficiency of the proposed protocols in Section 5, briefly describing our implemented eBook transaction system including its average execution time for main operations such as eBook registration, purchase, eBook download and storage; we analyze the system according to the size of eBook content and the level of difficulty used in the blockchain generation. Finally, we conclude our paper in Section 6.

## Related work

Peer-to-peer (P2P) systems allow all peers to distribute and share data directly with each other by willingly contributing their resources. Since year 2003, P2P file sharing traffic has surpassed web traffic and has been continuously grown up. Many P2P systems including Napster, Gnutella, BitTorrent, and Skype have been developed and still widely used [22]. Because various types of copyrighted digital content are easily distributed and shared among peers on the network, many copyright protection technologies have been proposed to prevent the illegal distribution of copyrighted content to unauthorized users. Most copyright protection technologies exploit digital watermarking [23–25] and fingerprinting technologies [26–28]. Digital watermarking embeds a watermark into the content that can be used to check the source of the content. The watermarking is classified into copyright watermarking and fingerprinting watermarking. The copyright watermarking inserts the copyright holder’s identification into the content in order to declare the copyright holder. But it cannot be used to trace an illegal distributer. On the other hand, the fingerprinting watermark insert a unique user identification into the content so that it can be used to track an illegal distributer [29]. A buyer-seller watermarking schemes [30–32] that incorporates both watermarking and fingerprinting mechanisms to protect the rights of both the buyer and the seller have been proposed. All of the above techniques are useful for detecting content owners or tracking illegal distributors, but do not prevent illegal distribution itself because the content copy holders can possess the same original contents in their local devices.

When accessing data (or content) stored on a remote peer or server, the integrity of the data should be verified first. That is, it is required to make sure that the remote server possesses the original data. There are three ways to prove the integrity of data stored in a remote server. The first is to check the hash value of data using the message authentication code (MAC) algorithm [33] or the hash function [34]. A verifier (or client) calculates the hash value of the original data and compares it with the hash value given from the remote server or with a previously calculated hash value. The main drawback of this approach is that it needs to access the entire data to calculate the hash value. The second approach is to use a third-party auditor (TPA) [35], which carries out all auditing process. On behalf of the data owner, the TPA issues an audit message (or challenge) to the remote server to validate the integrity of the data stored in the remote server. Any modification to the data has been occurred, the data owner is notified about those changes and then validates his or her data. The disadvantages of this method are the need for the third party as well as the communication channel with the third party. The last is the provable data possession (PDP) [36] model that generates probabilistic proofs of possession by sampling random sets of blocks from the server without retrieving the original data. Data is divided into small blocks and stored in remote server. Using with a homomorphic verifiable tag, a data owner generates a random challenge for specific data blocks, then the server responds with the proofs of possession of the blocks. The owner can validate the integrity of his or her data by verifying the validity of the proof without retrieving the original data or accessing to the entire data.

With the emergence of blockchain technology [6–15], publica [37]—a peer-to-peer publishing platform based on blockchain technology—was recently introduced; it enables authors to publish their works and allows direct transactions between readers and authors. A reader purchases a book token, which is an access key to a particular book, with cryptocurrency, and all purchase contracts are managed by blockchain. All works are stored in decentralized book storage, and readers with valid book tokens can download the books to their devices. However, the system does not allow for buyers to verify the validity of a purchased work or book token during the purchase. As a trusted party, Publica (or a publisher associated with the book) is responsible for the validity of the book content and the book token. However, in self-publishing in a trustless environment, readers must validate the content they have purchased and the corresponding access keys during the purchase. In addition, it is necessary to strictly prevent illegal copying and redistribution of downloaded eBook contents.

## Elliptic curve cryptography

We first describe the public-key cryptographic schemes, based on elliptic curve cryptography, that provide basic security in our system.

**(1) Elliptic curve over *F*_*p*_**

Let *F*_*p*_ be a prime finite field so that *p* is an odd prime number, and let *a*, *b* ∈ *F*_*p*_ satisfy the following Eq (1).

$$4∙a^{3}+27∙b^{2}≢0\;(modp)$$ (1)

An elliptic curve *E*(*F*_*p*_) over *F*_*p*_, which we use for our basic public-key cryptosystem (encryption and digital signature), consists of the set of points *P* = (*x*, *y*) for *x*, *y* ∈ *F*_*p*_ to the Eq (2).

$${E:y}^{2}\equiv x^{3}+a∙x+b\;(modp)$$ (2)

together with an extra point *O* called the point at infinity. For a given point *P* = (*x*, *y*), *x* is called the *x*-coordinate of *P*, and *y* is called the *y*-coordinate of *P*. The number of points on *E*(*F*_*p*_) is denoted by #*E*(*F*_*p*_), an additive Abelian group. Suppose that *P* = (*x*_1_, *y*_1_) and *Q* = (*x*_2_, *y*_2_) are points in *E*(*F*_*p*_): The following addition rules are defined:

- *O* + *O* = *O*
- *P* + *O* = *O* + *P* = *P*
- *P* + (–*P*) = *O*, where–(*x*, *y*) = (*x*,–*y*)
- If *x*_1_ ≠ *x*_2_, *P* + *Q* = (*x*_3_, *y*_3_) where

$$x_{3}\equiv \lambda^{2}-x_{1}-x_{2}\;(modp),y_{3}\equiv \lambda^{2}∙(x_{1}-x_{3})-y_{1}\;(modp),\;\mathrm{and}$$

$$\lambda \equiv \left\{ \begin{array}{c} \frac{y_{2}-y_{1}}{x_{2}-x\_1}\;(modp)\;P\neq Q\\ \frac{3∙x_{1}^{2}+a}{2∙y_{1}}\;(modp)\;P=Q \end{array}\right.$$

The domain parameters of *E*(*F*_*p*_) are denoted as *T* = (*p*, *a*, *b*, *G*, *n*, *h*) consisting of an integer *p* specifying the finite field *F*_*p*_, two elements *a*, *b* ∈ *F*_*p*_ specifying the above elliptic curve, a base point *G* = (*x*_*G*_, *y*_*G*_) on *E*(*F*_*p*_), a prime *n* that is the order of *G*, and an integer *h*, which is the cofactor *h* = #*E*(*F*_*p*_)/*n*.

**(2) Elliptic curve key pair**

For a given elliptic curve, an elliptic curve private-and-public key pair (*K*^−^, *K*^+^) = (*d*, *Q*) is generated as follows:

1. randomly select an integer *d* in the interval [1, *n*-1]
2. calculate *Q* = *dG*
3. output (*d*, *Q*)

**(3) Elliptic curve integrated encryption scheme (ECIES)**

For a given domain parameter *T*, let two users be *U* and *V*, and let the private-and-public key pairs of *U* and *V* be (*K*_*u*_^−^, *K*_*u*_^+^) and (*K*_*v*_^−^, *K*_*v*_^+^), respectively. Suppose that *U* sends a message *M* encrypted with *V*’s public key. The encryption algorithm is performed as follows:

1. select a random secret integer *k* and generated a point *R* = *kG* = (*x*_*R*_, *y*_*R*_)
2. compute *Q* = *k*·*K*_*v*_^+^ (= *k*·*d*_*V*_·*G*) = (*x*_*Q*_, *y*_*Q*_) and *z* = *x*_*Q*_
3. derive a symmetric encryption key and a MAC key *k*_*E*_ | *k*_*M*_ = *KDF*(*z*), where *KDF*(·) is the key derivation function
4. compute *EM* = *E*(*k*_*E*_, *M*) and *D* = *MAC*(*k*_*M*_, *EM*), where *E*(·) is a symmetric encryption algorithm and *MAC*() is a *MAC* scheme
5. output a ciphertext *C* = *R* | *EM* | *D*

The decryption algorithm of *V* for a ciphertext *C* is performed as follows:

1. compute *Q* = *K*_*v*_^−^·*R* (= *d*_*V*_·*k*·*G* = *k*·*d*_*V*_·*G* = *k*·*K*_*v*_^+^) = (*x*_*Q*_, *y*_*Q*_) and *z* = *x*_*Q*_
2. derive a symmetric encryption key and a MAC key *k*_*E*_ | *k*_*M*_ = *KDF*(*z*)
3. compute *MAC*(*k*_*M*_, *EM*), and if *MAC*(*k*_*M*_, *EM*) ≠ *D*, output ‘failed’
4. decrypt *M* = *D*(*k*_*E*_, *EM*) where *D*(·) is a symmetric decryption algorithm

**(4) Elliptic curve digital signature algorithm (ECDSA)s**

For the given domain parameter *T* and the private-and-public key pairs of two users *U*, *V* same as above, the signing operation of *U* for a message *M*, denoted as *Sig*(*K*_*u*_^−^, *M*), is performed as follows:

1. select a random secret integer *k* and generated a point *R* = *kG* = (*x*_*R*_, *y*_*R*_)
2. *r* = *x*_*R*_ mod *n*, if *r* = 0, return to step 1)
3. *e* = an integer conversion of *H*(*M*), where *H*(·) is a cryptographic hash function. If ⌈log_2_*n*⌉<8 (hash length), then *e* = an integer conversion of the leftmost ⌈log_2_*n*⌉ bits of *H*(*M*)
4. *s* ≡ *k*^-1^·(*e* + *r*·*K*_*u*_^−^) (mod *n*)
5. output (*r*, *s*)

The verifying operation *V* for a signature *S* = (*r*, *s*) is performed as follows:

1. *e* = an integer conversion of *H*(*M*), if ⌈log_2_*n*⌉<8 (hash length), then *e* = an integer conversion of the leftmost ⌈log_2_*n*⌉ bits of *H*(*M*)
2. *u*_1_ ≡ *e*·*s*^-1^ (mod *n*) and *u*_2_ ≡ *r*·*s*^-1^ (mod *n*)
3. compute *R* = (*x*_*R*_, *y*_*R*_) = *u*_1_*G* + *u*_2_*K*_*u*_^+^. If *R* = *O*, then output ‘invalid’
4. *v* ≡ *x*_*R*_ (mod n). If *v* = *r*, output ‘valid’, otherwise, output ‘invalid’

## Blockchain-based eBook transaction system

### System configuration, assumptions and security requirements

The proposed eBook transaction system consists of user, service application, eBook content, digital coin, blockchain, book repository, and P2P network. Users can be either authors or readers, and each user can use all the services of the proposed system only through the service application; the service application, denoted as *SA*, is dedicated software that conducts all functions specified in our system. We give a detailed description of the *SA* in the next subsection. An eBook is defined as a digital text document that can be sold independently, and it can be a stand-alone book or part of a series; the digital coin in the proposed system is a cryptocurrency used to pay for eBook purchases. Existing cryptocurrencies such as Bitcoin [6–7] and Ethereum [8] can be used, but our implemented service application used our developed cryptocurrency. All eBook information and eBook purchase transactions are distributed over the network and maintained on blockchains named *B_Chain* and *C_Chain*, respectively. A book repository, which acts as a content server, stores all published eBook contents in an encrypted manner. Every eBook is divided into small pieces of book content, and each piece of book is encrypted with its own book key. The repository stores only the encrypted pieces of eBooks but does not store any private or secure information including book keys; the repository also has an authentication logic layer that verifies whether an upload or download request is valid or not.

**Fig 1:**
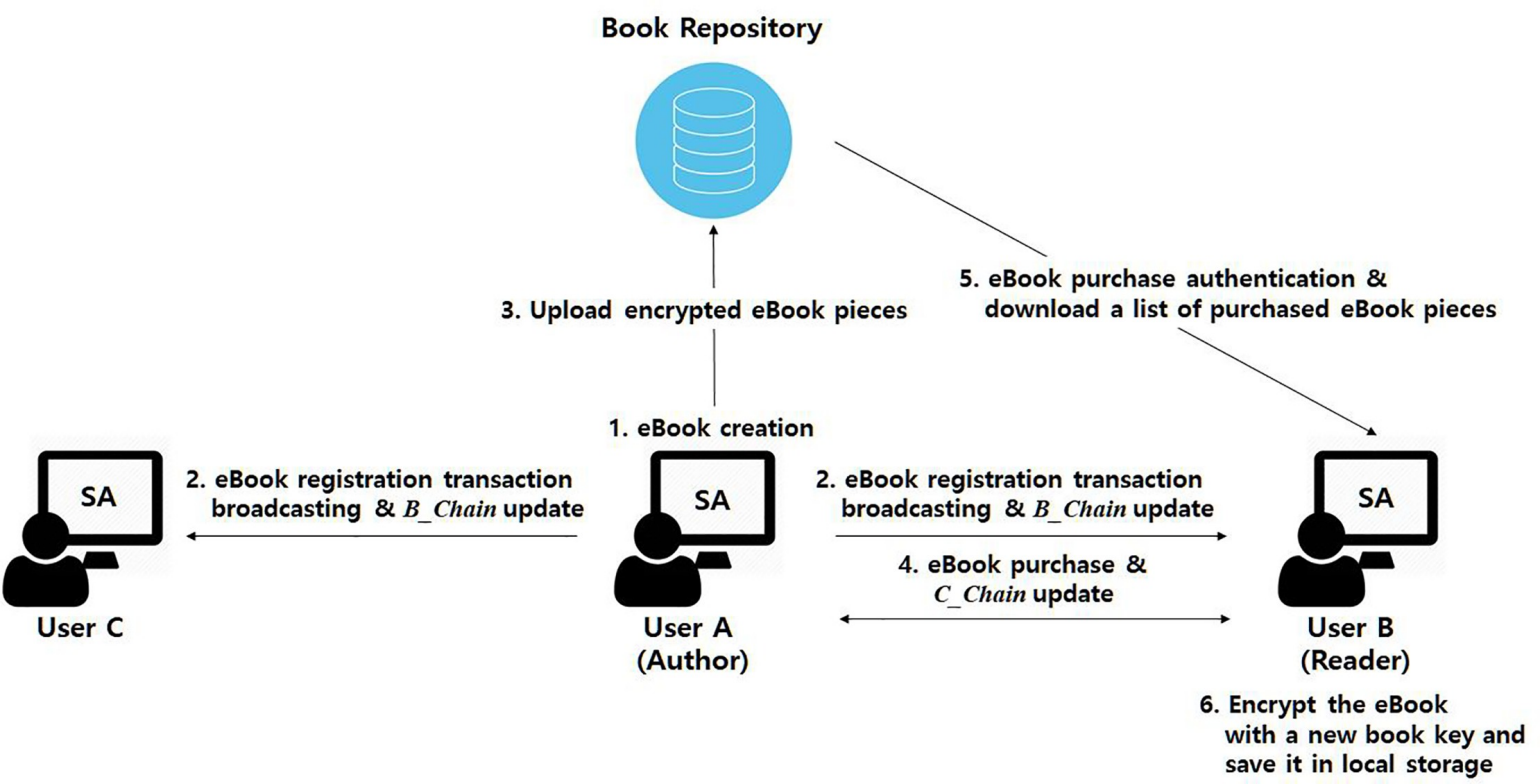
eBook transaction system configuration and the operation of eBook publishing and purchasing.

Fig 1 shows the configuration of the proposed system and an illustrated scenario of eBook registration and purchase. Any user can either publish a new eBook using a *SA* or purchase and read an eBook using the application. Once an author *A* completes a new book, *A* registers the book. The book information is broadcast to all users on the network, and *B_Chain* is updated. Encrypted pieces of the book are uploaded to the book repository; the current book list of every *SA* is also then updated. If a reader *B* wants to buy *A*’s published book, *A* and *B* complete that purchase protocol between them; *B* can validate the book before completing the purchase with a book key obtained during the purchase process. When *A* and *B* complete a valid transaction, the transaction deploys on the network, and *C_Chain* is updated. *B* can request the encrypted pieces of the book from the book repository, which then validates the purchase transaction and *C_Chain*. If all are valid, *B* downloads the book pieces, re-encrypts them with a new key, and stores them locally.

For practical use of the proposed system, the following assumptions are made:

- The *SA* is trusted. The provider of the proposed system is responsible for keeping the system available and safe, and for resolving any claims incurred while using the proposed system. But, the provider is not responsible for the published content itself.
- The *SA* has a master secret key programmed in a secure manner, and it is impossible for users to know the master key.
- Only eBook purchase is allowed. eBook rental, which grants a right to read for a particular period, is disallowed.
- Only authors have the ownership of their created eBook contents; eBook readers have only a “right to read” for their purchased contents. Thus, readers cannot resell their purchased books.
- The proof of working (or mining block) for a blockchain update can be accomplished by any user on the network, but for efficient and practical use of the scheme, the author is basically responsible for the proof of work.

**Fig 2:**
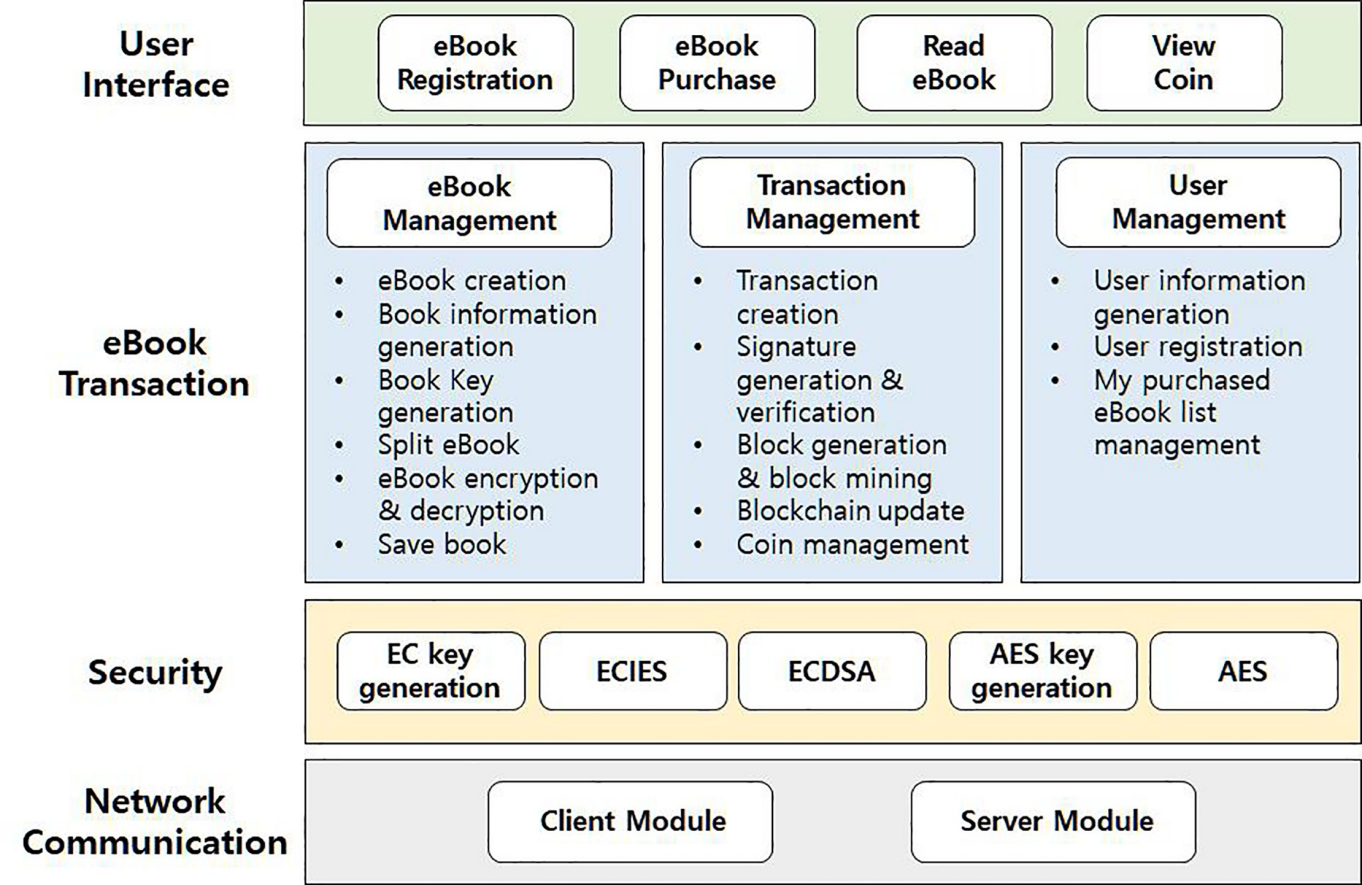
The configuration of a service application.

*SA* is the most crucial element in our system; Fig 2 shows the *SA* configuration. *SA* consists of four main modules: communication, security, eBook transaction, and user interface. The communication module is responsible for all kinds of P2P communications between users on the network including the book repository. The security module is responsible for performing fundamental cryptographic protocols including public key encryption and decryption, digital signature and verification, and symmetric key encryption and decryption. SHA256 [38–39] is used for a hash algorithm, and ECDSA is used for a basic digital signature algorithm. ECIES is used for a public key encryption and decryption algorithm, and AES [40–41] with the key size of 256 bits is used for a symmetric key encryption and decryption algorithm. The eBook transaction module, which plays a key role in the proposed system, performs three main actions: eBook management, transaction management, and user management. The eBook management relates to when a new eBook is created, generates its book key, divides the book into the pieces and encrypts them, and so on. The transaction management creates blocks for eBook registration transactions and eBook purchase transactions, updates the corresponding blockchain, and manages users’ digital coin. The user management module registers users and managers their book lists. Finally, the user interface module provides all kinds of user interfaces for eBook registration, purchase, reading, and showing current coin.

Next, we describe the format of transactions for eBook registration and eBook purchase, and explain the blockchain update process. The format of both types of transactions are given in Table 1.

**Table 1:** Format of transactions for eBook information registration and eBook purchase.

(a) The format of eBook registration transaction		(b) The format of eBook purchase transaction	
Field	Description	Field	Description
**ID**	The hashed result of this transaction	**ID**	The hashed result of this transaction
**Signature of author**	The author’s digital signature on the transaction content	**Signature of buyer**	The buyer’s digital signature on the contract information
**Content**	The eBook information	**Signature of author**	The author’s digital signature on the contract information
		**Contract**	The detailed eBook purchasing information

A new block containing eBook registration transactions collected during a predefined time interval is periodically created, and the block is added to the current *B_Chain*. In contrast, in order to enable real-time eBook purchases, a new block containing the purchase transaction is created whenever a new purchase transaction occurs, and the block is added to the current *C_Chain*. Originally, the hash value of the new block is determined as the root hash value of the Merkle tree composed of the hash values of transactions included in the block. For *C_Chain*, because a block usually contains a single transaction, the transaction hash value becomes the block’s root hash value.

As assumed above, every author is supposed to generate the proof-of-work about a current block. The proof-of-work for each block is to find a nonce that can generate a hash value starting with 0 as many as the number specified in a predefined difficulty field. The format of each block is given in Table 2.

**Table 2:** Format of block.

Field	Description
**Block hash**	The hash value of this block
**ID**	The sequence number of this block
**Previous block hash**	The hash value of the previous block in the chain
**Merkle tree root**	The root hash value of the Merkle hash tree
**Timestamp**	The creation time of the block in the chain
**Difficulty**	The level of difficulty for the proof-of-work
**Nonce**	A nonce for the proof-of-work
**Transactions**	All transactions in the block

The algorithm for updating blockchain *add*(*chain*, *transaction*) and the algorithm for verifying transaction *verify*(*chain*, *transaction*, *block_id*) are given below:

*add*(*chain*, *transaction*) performs

1. *TID*_*i*_ = a set of hash values of transactions collected for the *i*-th time interval;
2. compute *tid* = *H*(*transaction*);
3. if (verify the *signature* of *transaction*) then *TID*_*i*_ = *TID*_*i*_ ∪ {*tid*};
4. if (block creation time) then
  - p*revious_block_hash* = *block_hash* of *chain*;
  - compute *root_hash* = *merkle_tree_root_hash*(*TID*_*i*_);
  - find *nonce* such that *H*(*previous_block_hash* | *nonce* | *root_hash*) satisfies the difficulty;
  - generate a new *block* and update *chain* = *H*(*previous_block_hash* | *nonce* | *root_hash*);

*verify*(*chain*, *transaction*, *block_id*) performs

1. find *block*_*id*_ with *block_id* in *chain*;
2. compute *tid* = *H*(*transaction*) and find a transaction *T*_*id*_ with *tid* in *block*_*id*_;
3. obtain a public key *pk* from *contract* of *T*_*id*_;
4. if (verify the *signature* of *transaction* with *pk*)
  - generate *new_TID* = *TID*–{*tid*_*id*_} ∪ {*tid*} where *tid*_*id*_ is the hash of *T*_*id*_;
  - compute *new*_*root_hash* = *merkle_tree_root_hash*(*new*_*TID*);
  - *previous_block_hash*_*id*_ = *previous_block_hash* of *block*_*id*_;
  - *nonce*_*id*_ *= nonce* of *block*_*id*_;
  - compute *new*_*block_hash* = *H*(*previous_block_hash*_*id*_ | *nonce*_*id*_ | *new*_*root_hash*);
  - if (*new*_*block_hash* = *block_hash* of *block*_*id*_) output *true*;

else output *false*;

The main goal of the proposed system is to provide a secure and reliable eBook transaction system. Thus, we designed our system to satisfy the following security requirements:

- **Ownership**: It is possible to identify the owner of each eBook. It is impossible to forge or modify the owner of the eBook without knowing the private key of the owner.
- **Authentication**: It is possible to authenticate if a user is a legitimate purchaser.
- **Authorization**: It is possible to authorize if a user has a right to read a particular eBook’s content. Only a legitimate purchaser who has a valid purchase transaction recorded in the blockchain has a right to read the purchased book.
- **Confidentiality of digital content**: It is impossible to obtain unencrypted and original eBook contents without knowing the book keys of the contents.
- **Non-forgeability of digital content**: It is impossible for a non-author to forge or modify eBook contents that have been already published.
- **Verifiability of digital content**: It is possible to verify the author and content validity of an eBook.
- **Readability**: The eBook contents can be restored only at the service application. It is impossible to read the contents in other ways.
- **Non-forgeability of purchase transaction**: It is infeasible to forge any eBook purchase transaction.
- **Non-repudiation of purchase transaction**: It is infeasible to repudiate previously performed purchases.
- **Verifiability of transaction**: Every eBook purchase transaction is verifiable.
- **Forbidden of piracy and illegal distribution**: Only authors have the ownership of their published eBook contents. A non-author can neither distribute nor resell the purchased book.

We assume the threat model of malicious users as follows: Malicious users can attempt to

1. upload content that does not make sense, such as a compilation of meaningless texts;
2. manipulate contents that are purchased and stored in their local devices and distribute or upload the fabricated or copied contents;
3. download contents from the book repository without valid purchase transactions;
4. stop the purchase process abnormally before the purchase process completes.

The main notations used in the proposed eBook transaction protocols are summarized in Table 3.

**Table 3:** Notations.

Notation	Description
***U***_***i***_	The *i*-th user
***ID***_***i***_	The ID of *U*_*i*_
***PW***_***i***_	The password of *U*_*i*_
$<K_{i}^{+},K_{i}^{-}>$	The public and private key pair of *U*_*i*_
***SA***_***i***_	The service application of *U*_*i*_
***MK***	The master secret key of service application
$<AK_{i}^{+},{AK}_{i}^{-}>$	The public and private key pair of *SA*_*i*_
**<*BK***^**+**^**,*BK***^**−**^**>**	The public and private key pair of the book repository
***B***_***j***_	The *j*-th e-book of *U*
***BID***_***j***_	The book ID of *B*_*j*_
***bk***_***j***_	A symmetric book key for *B*_*j*_
***PKE*(*K***^**+**^**,*M*), *PKD*(*K***^**−**^**,*C*)**	A public key encryption and decryption algorithms (ECIES) with a public and private key pair <*K*^+^,*K*^−^>
***E*(*k*,*M*), *D*(*k*,*C*)**	A symmetric key encryption and decryption algorithms (AES) for with a symmetric key *k*
***H*(*M*)**	A cryptographic hash function (SHA256)
***Sig*(*K***^**−**^**,*M*)**	A digital signature algorithm (ECDSA) for a message *M* with a private key *K*^+^.
***verify*(*chain*,*transaction*,*block_ID*)**	A blockchain algorithm to verify the validity of a transaction in the chain
***add*(*chain*,*transaction*)**	A blockchain algorithm to add a new transaction to current blockchain

### The proposed eBook trading protocols

#### User registration

All eBook services are available by installing the service application. Every *SA* has the same master secret key denoted as *MK* that has been already programmed in the application in a secure manner. Let *SA*_*i*_ be the service application of a user *U*_*i*_. The installed *SA*_*i*_ initially performs a user registration process, which produces *U*_*i*_’s ID (*ID*_*i*_), password (*PW*_*i*_), and public-and-private key pair (*K*_*i*_^+^ and *K*_*i*_^−^) and *SA*’s public-and-private key pair (*AK*_*i*_^+^ and *AK*_*i*_^−^); *U*_*i*_ chooses its own ID, password, and public-and-private key pair. The user’s private information is encrypted with *SA*’s private key, and *SA*’s private key is encrypted with *MK*. Thus, it is impossible for any entity except for the *SA* itself to know the *SA*’s private key in any way. All private information is stored in *U*_*i*_’s local storage as encrypted. Then, user information, denoted as *user_info* (user’s ID, user’s public key, and *SA*’s public key), is generated as follows:

$$user\_info=“ID_{i}\left|K_{i}{}^{+}\right|AK_{i}{}^{+}”$$ (3)

**Fig 3:**
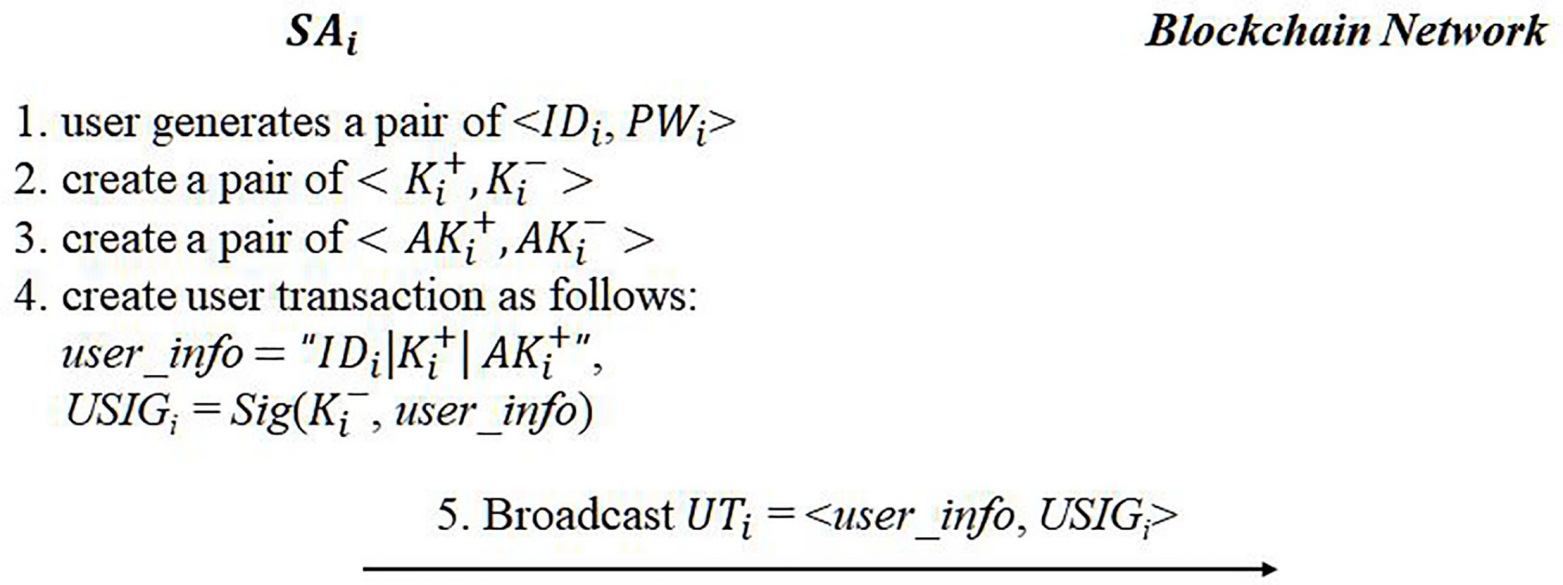
User registration.

*user_info* is broadcast with its signature *USIG*_*i*_ = *Sig*(*K*_*i*_^−^, *user_info*). All *SA*s over the network add the new user to their user lists. The user registration process is given in Fig 3:

The *SA* requires a user’s password to authenticate the user, simply checking the validity of the password given whenever a user utilizes his or her *SA*.

#### eBook registration

Any user can upload his or her digital content through the eBook management module of *SA*. eBook registration consists of two steps: updating *B_Chain* with new book information and uploading encrypted eBook content to the book repository. Let the *j*-th digital content of an author *U*_*A*_ be *B*_*j*_. *SA*_*A*_ first chooses a random secret key *s*_*j*_ and creates a book key *bk*_*j*_ of *B*_*j*_ as follows:

$$bk_{j}=H\left(s_{j}|MK\right)$$ (4)

The reason the master secret key of *SA*_*A*_ is used to generate the book key is so that only the legitimate *SA*, which knows the master secret key, can produce the same book key later.

*B*_*j*_ is divided into *n* pieces of the same size except for the last piece, and these are denoted as *B*_*j*_ = {*b*_*j*,1_| *b*_*j*,2_|…| *b*_*j*,*n*_}; the size of the last piece is less than or equal to that of the other pieces. The number of pieces depend on the book size. *SA*_*A*_ generates a hash value of *B*_*j*_ and hash values of individual file piece *b*_*j*,*k*_, and then produces a hash list for all file pieces, such as *H*_*j*_ = *H*(*B*_*j*_) and *HL*_*j*_ = {*H*(*b*_*j*,1_)|*H*(*b*_*j*,2_)| … |*H*(*b*_*j*,*n*_)}. In order to protect the intellectual property of an eBook, the original content has to be encrypted; that is, there is no way for any buyer to check if the buying content is valid until the purchased is completed. A buyer can obtain only encrypted content and its corresponding book key after completing a purchase; if the revealed eBook content is meaningless or garbage, the buyer loses the purchase price. In contrast, we believe that a buyer should be able to verify an eBook’s content during the purchase process. Our strategy is to allow a buyer to acquire a random piece of the book during the purchasing process to check its validity, and if that random piece is valid, the buyer continues the purchasing process.

*SA*_*A*_ encrypts each piece with the book key. The completely encrypted content is *CB*_*j*_ = {*E*(*bk*_*j*_, *b*_*j*,1_)| *E*(*bk*_*j*_, *b*_*j*,2_)|…| *E*(*bk*_*j*_, *b*_*j*,*n*_)}, and this generates *U*_*A*_’s digital signature for *CB*_*j*_, which is *SB*_*j*_ = *Sig*(*K*_*A*_^−^, *CB*_*j*_ | *HL*_*j*_). *SA*_*A*_ generates book information as follows:

$$BID_{j}=H(book\_title|ID_{A}|K_{A}{}^{+}),$$ (5)

$$book\_info=“BID_{j}\left|book\_title\right|ID_{A}\left|K_{A}{}^{+}\right|H_{j}\left|book\_price\right|SB_{j}\left|summary_{j}\right|timestamp”,$$ (6)

where *BID*_*j*_ is the ID of *B*_*j*_, *ID*_*A*_ is the author’s ID, and *K*_*A*_^+^ is the author’s public key. *summary*_*j*_ is the summary of the book for preview. A pair of *HL*_*j*_ and *CB*_*j*_ is stored in a separate book repository, and only legal book purchasers can obtain those. The author’s signature *SB*_*j*_ for *CB*_*j*_ and *HL*_*j*_ is used in the eBook upload to the book repository. The book repository can use the author’s signature to verify that the author is uploading given content.

The author’s signature for *book_info* is *BSIG*_*j*_ = *Sig*(*K*_*A*_^−^,*book_info*), and a final book registration transaction denoted as *BTRS*_*j*_ is broadcast as follows:

$$Transaction_{j}=“BSIG_{j}\left|book\_info”\;and\;BTRS_{j}=“H\left(Transaction_{j}\right)\right|Transaction_{j}”$$ (7)

**Fig 4:**
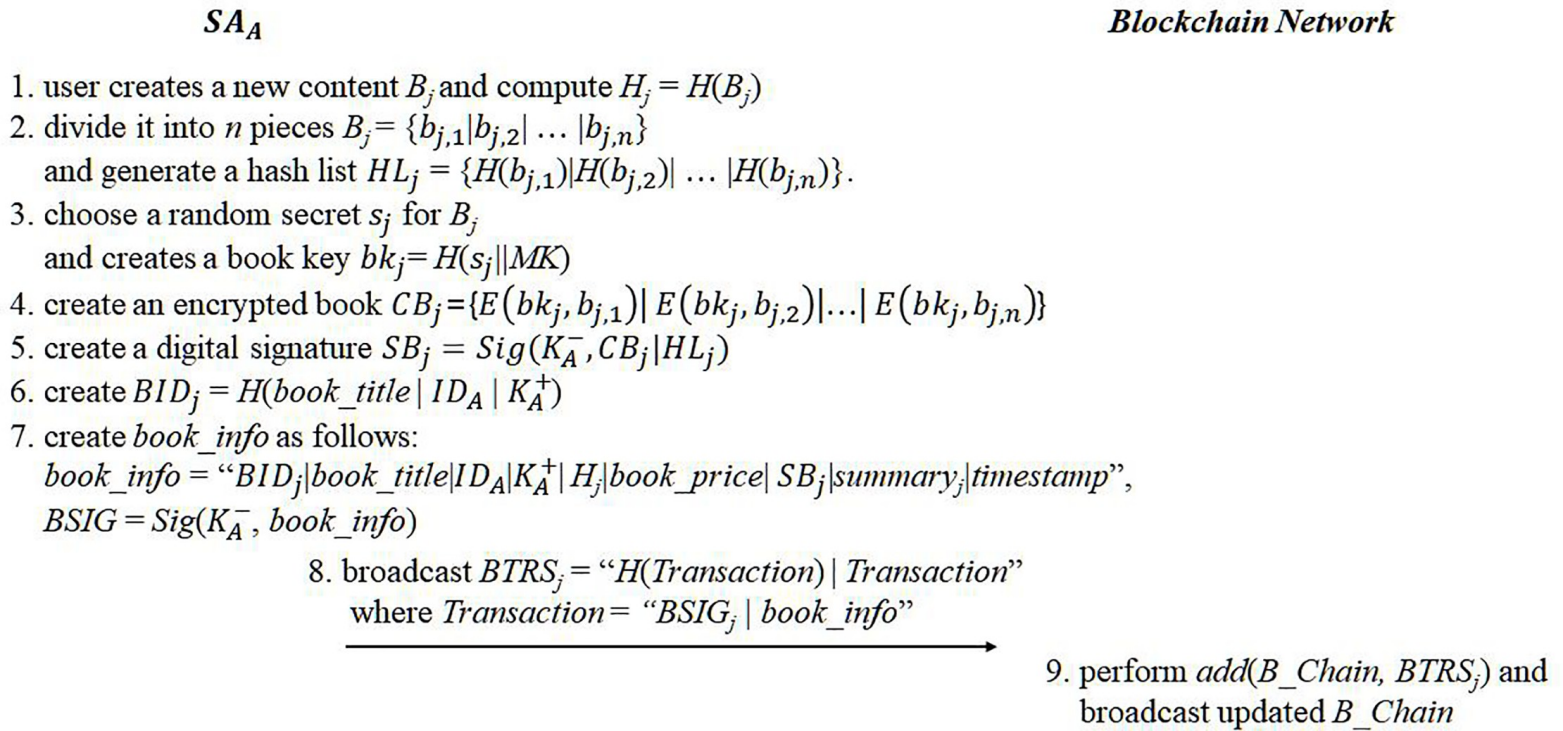
eBook registration.

At least, all *SA*s that generated *BTRS*s perform the proof-of-work for a block containing all *BTRS*s collected for a predefined period, and *B_Chain* is updated with the first generated block hash value. Since the updated *B_Chain* is deployed over the entire network including the book repository, every online *SA* updates their book lists to include all the new book information. Fig 4 summarized the eBook registration protocol.

As *B_Chain* is updated, *SA*_*A*_ uploads the encrypted contents and hash list to the book repository. Suppose that the book repository possesses all *BTRS*s recorded on current *B_Chain*. *SA*_*A*_ generates book-upload request message *book_upload* = “*block_ID* | *BTRS_ID* | *BID*_*j*_ | *timestamp*” and its signature *BUSIG* = *Sig*(*K*_*A*_^−^, *book_upload*). Also, *SA*_*A*_ encrypts “*CB*_*j*_ | *HL*_*j*_” with the book repository’s public key. Finally, *SA*_*A*_ sends the following book-upload request, *BURQST*, to the book repository:

$$BURQST=<book\_upload,BUSIG,\;PKE\left(BK^{+},\;CB_{j}|HL_{j}\right)>$$ (8)

*block_ID* is the sequence number of the block that contains the *BTRS* of the uploaded eBook. *BTRS_ID* indicates the *ID* field of the *BTRS*. The book repository reveals *CB*_*j*_ | *HL*_*j*_ with its private key and finds the corresponding *BTRS* with *BTRS_ID* in the block. It obtains *BID*_*j*_, *K*_*A*_^+^ and *SB*_*j*_ from *book_info* in the *BTRS* and verifies *BID*_*j*_, *BUSIG* and *SB*_*j*_ for the given *BID*_*j*_, *book_upload*, and the revealed *CB*_*j*_ and *HL*_*j*_. If the signatures are all valid, then, the contents are stored in the repository. Fig 4 presents the eBook registration protocol.

**Fig 5:**
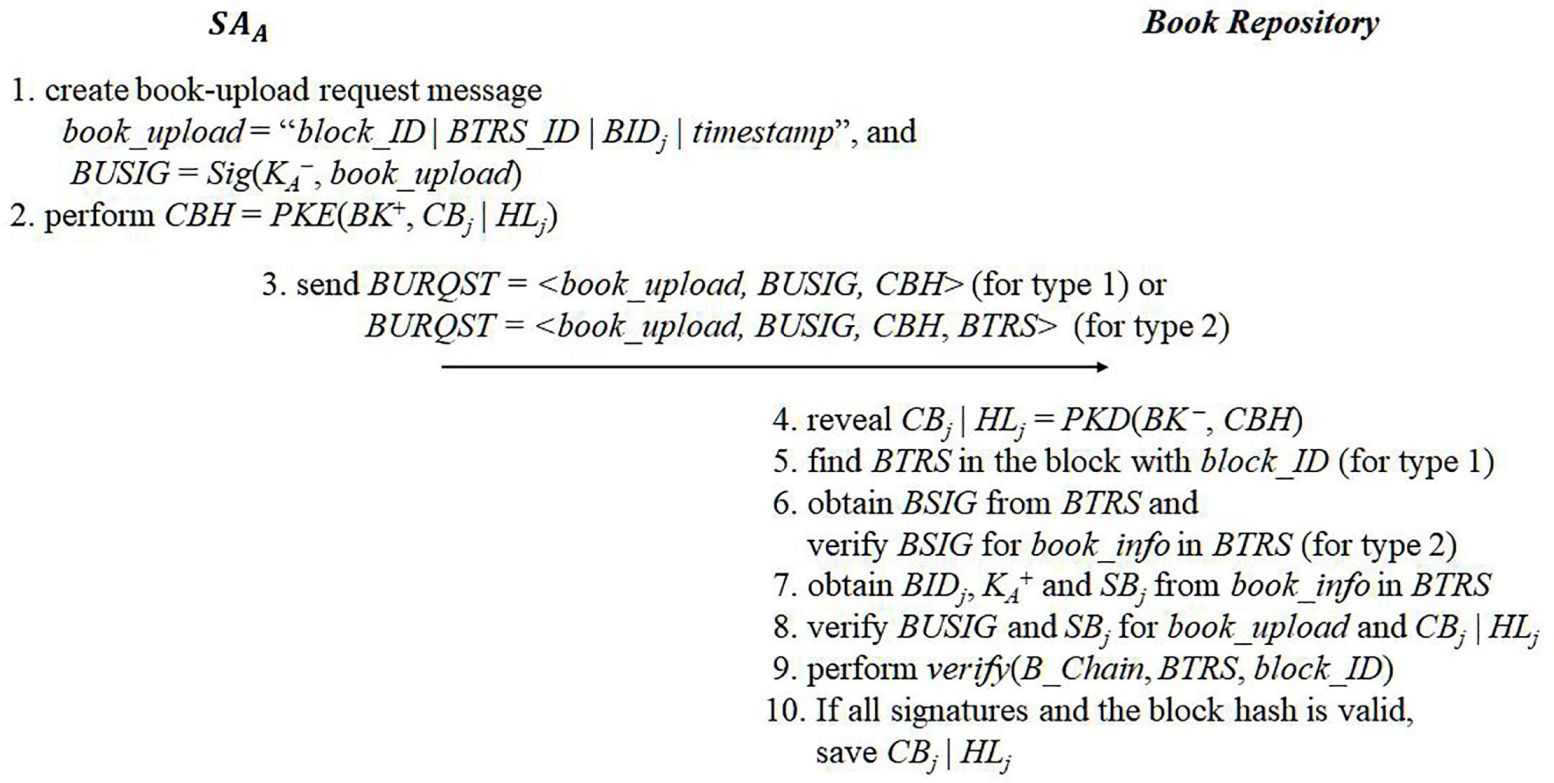
eBook upload.

Alternatively, the book repository can only store hash values of all *BTRS*s for saving the storage costs. In this case, *SA*_*A*_ sends the *BTRS* of the uploaded book along with *BURQST*. In addition to perform the same verification process for the *BURQST*, the book repository needs to validate the *BTRS* itself. It computes the hash value of *BTRS*, the Merkle tree root hash for all *BTRS*s in the block, and the block hash. If the computed block hash is identical to the block hash of *B_Chain*, then the contents are stored in the repository. Fig 5 shows the eBook upload protocol.

#### eBook purchase

A user pays a fee to the author directly for the right to read an eBook. Thus, the main goal of the purchase process is to securely provide a buyer with a valid book key through a validated eBook purchase contract between the author and the buyer. Before making the agreement, the buyer should be able to validate both the purchased book’s content and the received book key. Also, the author must receive the buyer’s confirmation that the buyer has obtained a valid book key. To achieve this, the two sides perform the following eBook purchasing process interactively.

Let *SA*_*R*_ and *SA*_*A*_ be the service application for a book reader and an author, respectively. *SA*_*R*_ first generates a contract request message *request* as follows:

$$CID=H\left(BID_{j}\left|ID_{A}\right|ID_{R}|timestamp\right),$$ (9)

$$request=“CID\left|BID_{j}\right|ID_{A}\left|ID_{R}\right|K_{R}{}^{+}\left|AK_{R}{}^{+}\right|book\_price\left|balance_{R}\right|rindex|timestamp”,$$ (10)

where *CID* is a new contract ID, *AK*_*R*_^+^ is *SA*_*R*_’s public key, *balance*_*R*_ is *U*_*R*_’s current coin balance, and *rindex* is a random number in the interval [1, *n*_*BP*_], where *n*_*BP*_ is the total number of pieces of the book. *rindex* is required to validate the content of a book for purchase. The reader’s private key digitally signs the request message as *RSIG*_*R*_ = *Sig*(*K*_*R*_^−^, *request*). The final contract request message *CRQST*_*R*_ = <*request*, *RSIG*_*R*_> is then broadcast.

Second, *SA*_*A*_ responds to *SA*_*R*_ with a contract message and a book key, and the book repository responds to *SA*_*R*_ with the encrypted book piece and its hash value corresponding to *rindex* specified in *request*. *SA*_*A*_ generates a contract message and its digital signature. *SA*_*A*_ replies with a message that includes an encrypted secret book key, the contract message, and its signature. The contract message, denoted as *contract*, is defined as follows:

$$contract=“CID\left|BID_{j}\right|ID_{A}\left|K_{A}{}^{+}\right|ID_{R}\left|K_{R}{}^{+}\right|AK_{R}{}^{+}\left|book\_price\right|nbalance_{A}\left|nbalance_{B}\right|timestamp”$$ (11)

*nbalance*_*A*_ is the updated balance of *U*_*A*_ after adding the book price to *U*_*A*_’s current balance, and *nbalance*_*R*_ is the updated balance of *U*_*R*_ after the book price is deducted from the *U*_*R*_’s current balance. The author’s private key digitally signs the contract message as *CSIG*_*A*_ = *Sig*(*K*_*A*_^−^, *contract*). *SA*_*A*_ encrypts a partial book key *s*_*j*_ with *SA*_*R*_’s public key as follows:

$$ck_{j}=PKE\left(AK_{R}{}^{+},s_{j}\right)$$ (12)

This is to allow for only a legal *SA*_*R*_ to produce a valid book key. The final contract reply message *CRPY*_*j*_ = < *ck*_*j*_, *contract*, *CSIG*_*A*_> is then broadcast.

The book repository chooses the encrypted book piece and hash value corresponding to *rindex* specified in the request message and encrypts it with *SA*_*R*_’s public key as well. Note that *SA*_*R*_’s public key is specified in *request*. The repository responds to *SA*_*R*_ with *cb*_*j*,*rindex*_ as follows:

$$cb_{j}{}_{,rindex}=PKE\left(AK_{R}{}^{+},E\left(bk_{j},\;b_{j}{}_{,rindex}\right)|HL_{j,rindex}\right)$$ (13)

Lastly, *SA*_*R*_ validates the given book key and the eBook content. If both the key and the content are valid, then *SA*_*R*_ broadcasts a confirm message digitally signed by the reader’s private key; otherwise, *SA*_*R*_ generates a fail message signed by the reader’s key. *SA*_*R*_ obtains the partial book key by decrypting *ck*_*j*_ with *SA*_*R*_’s private key as *s*_*j*_ = *PKD*(*AK*_*R*_^−^, *ck*_*j*_). Then, *SA*_*R*_ produces the original book key using its master secret key as *bk*_*j*_ = *H*(*s*_*j*_ | *MK*). Also, *SA*_*R*_ decrypts *cb*_*j*,*rindex*_ with its private key and obtains the encrypted book piece *E*(*bk*_*j*_, *b*_*j*,*rindex*_) and the corresponding hash value *HL*_*j*,*rindex*_. *SA*_*R*_ decrypts *E*(*bk*_*j*_, *b*_*j*,*rindex*_) with its generated book key *bk*_*j*_ and produces a hash value of *H*(*b*_*j*,*rindex*_). If the produced hash value is identical to the given value, then the book key is proven correct. In addition, it is proven that the contents stored in the book repository are the original contents that the author created and uploaded (that is, the content was not modified).

*SA*_*R*_ shows a fragment of the decrypted book piece *b*_*j*,*rindex*_ to *U*_*R*_. *U*_*R*_ checks if the decrypted content makes sense, that is, if it consists of meaningful text. If both the key and the content are valid, *SA*_*R*_ generates a digital signature for *contract CSIG*_*R*_ = *Sig*(*K*_*R*_^−^, *contract*) and generates and broadcasts a final purchase transaction, *CTRS*, as follows:

$$Transaction=“CSIG_{R}\left|CSIG_{A}\right|contract”\;and\;CTRS=“H\left(Transaction\right)|Transaction”$$ (14)

**Fig 6:**
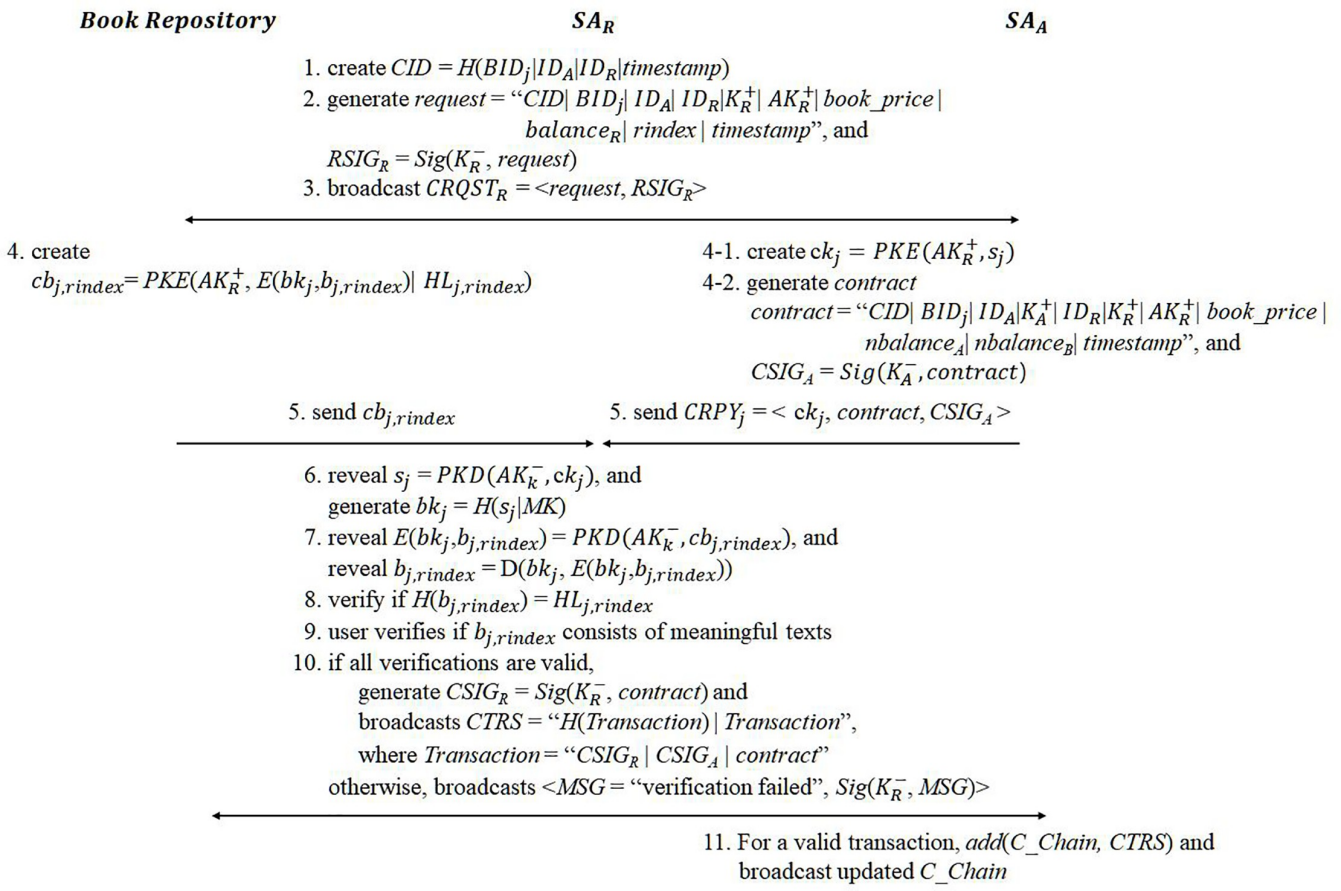
eBook purchase transaction.

Otherwise, *SA*_*R*_ broadcasts a fail message <*MSG* = “verification failed”, *Sig*(*K*_*R*_^−^, *MSG*)> and the purchase is terminated. For a valid transaction, *SA*_*A*_ just generates a block containing *CTRS* and adds it to *C_Chain*. *C_Chain* is broadcast to all users including the book repository, and *SA*_*R*_ and *SA*_*A*_ update their users’ coins. The book repository does not need to store all *CTRS*s but only keeps the transaction hash values, block nonce and the block hash. Fig 6 summarizes the eBook purchase protocol.

#### eBook download and storage

Once the contract has been completed successfully, *SA*_*R*_ can download the encrypted eBook pieces from the book repository. *SA*_*R*_ first generates a book request message with its transaction, and if the transaction is valid, then the repository responds with the encrypted book pieces. The book request message is constructed as *book_request* = “*block_ID* | *CTRS_ID* | *BID*_*j*_ | *timestamp*” and its signature is generated as *BRSIG* = *Sig*(*AK*_*R*_^−^, *book_request*). A final book request, *BRQST*, is sent to the book repository:

BRQST=<book_request,BRSIG,CTRS> (15)

**Fig 7:**
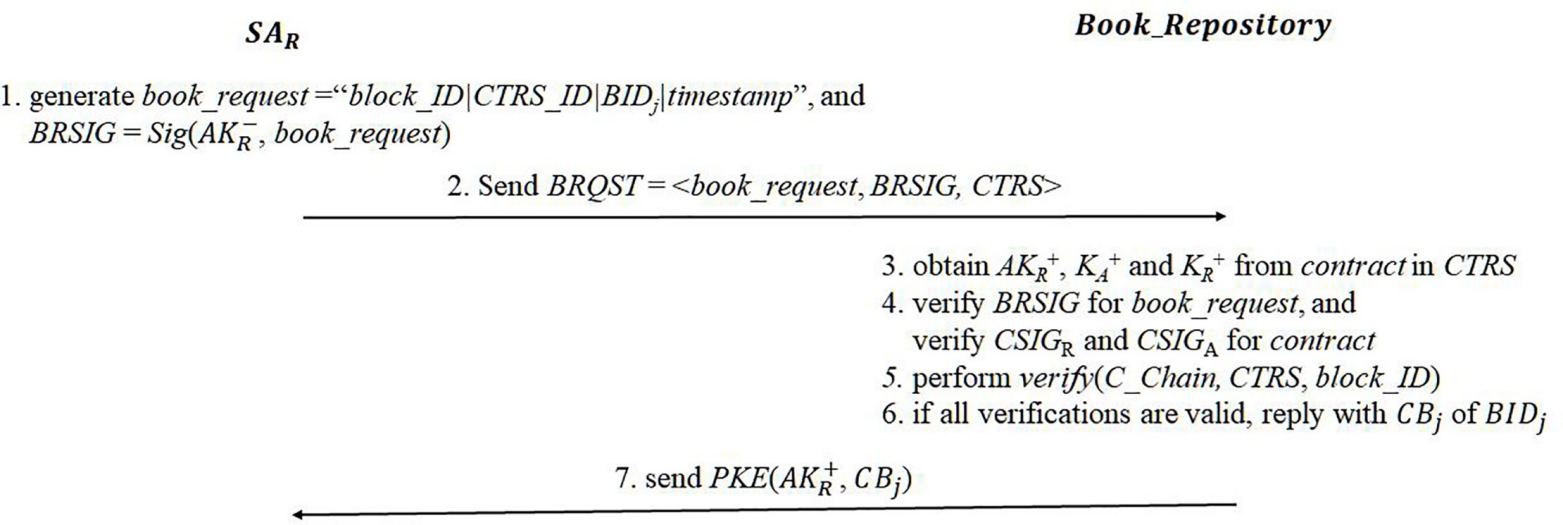
eBook download.

**Fig 8:**
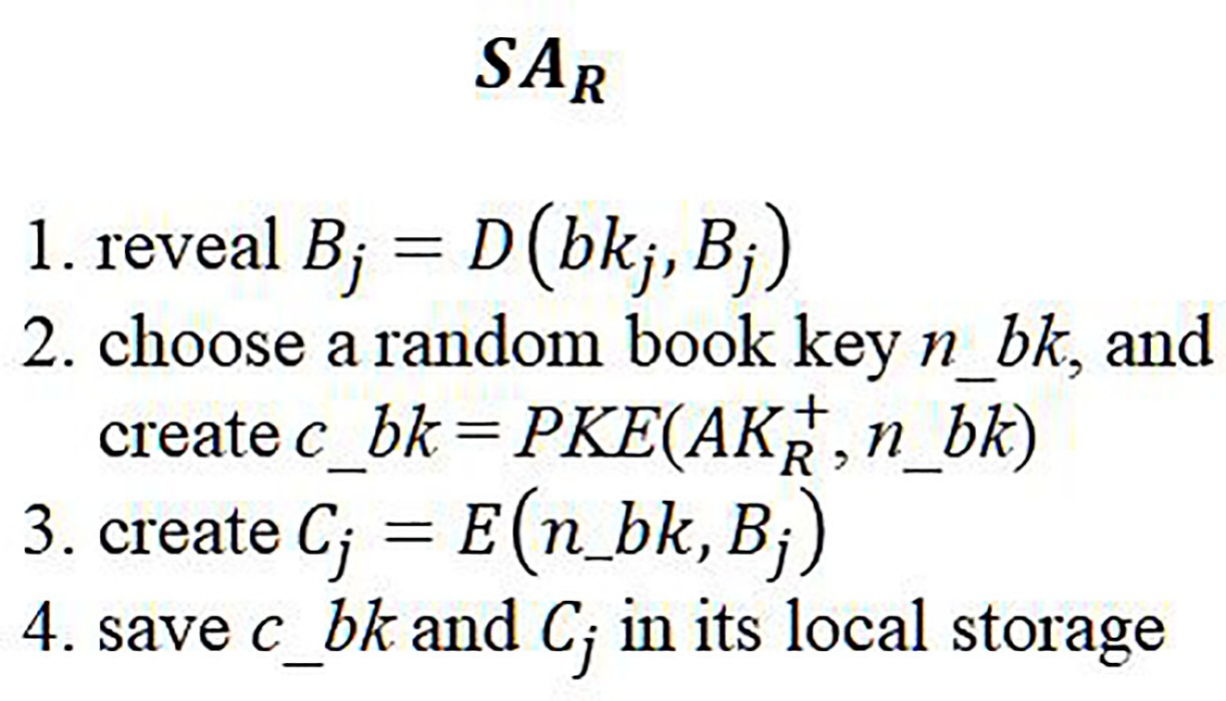
Re-encryption and save eBook.

*block_ID* is the sequence number of the block containing *CTRS*, and *CTRS_ID* indicates the *ID* of *CTRS*. The book repository obtains *AK*_*R*_^+^, *K*_*A*_^+^ and *K*_*R*_^+^ from *contract* in *CTRS*. It firstly verifies the validity of *BRSIG* with *AK*_*R*_^+^ and verifies two signatures *CSIG*_*R*_ and *CSIG*_*A*_ for *contract* with *K*_*A*_^+^ and *K*_*R*_^+^. If the signatures are all valid, then it computes the hash value of *CTRS* and its block hash. If the computed block hash is identical to the block hash in the *C_Chain*, then the transaction is validated. Then, the repository replies with *CB*_*j*_ encrypted with *AK*_*R*_^+^ as *PKE*(*AK*_*R*_^+^, *CB*_*j*_). Because a valid *C_Chain* is required to download the eBook, it is impossible to obtain the original eBook content without completing the purchase transaction, even though the book key has been obtained. The eBook download protocol is given in Fig 7:

Once the eBook has been downloaded, *SA*_*R*_ decrypts the original content of the book and re-encrypts it with *SA*_*R*_’s randomly chosen secret key, and then it stores the book in the reader’s local storage. Thus, even for the same eBook, each reader receives a differently encrypted version of the book for local storage. Additionally, each encrypted version can be decrypted only by *SA*. The detailed protocol is given in Fig 8:

Users can read books they have purchased legally through their *SA*. Whenever an eBook read request occurs, *SA* reveals the book key *n_bk* = *PKD*(*AK*_*R*_^−^, *c*_*bk*), reveals the book *B*_*j*_ = *D*(*n*_*bk*, *C*_*j*_), and shows the book through the *SA* viewing module. Because the purchased content is encrypted with a new book key and also with the *SA*’s public key, the user cannot obtain the original and unencrypted content from the local device. Because the encrypted book key can be restored only by the *SA*’s private key, the other *SA*s cannot restore the encrypted contents without knowing the *SA*’s private key, even if the user distributed the encrypted content to others.

### Additional remarks

We explain further some distinct features of the proposed system by answering the following three questions.

**(1) Why is the master secret key of SA required?**

Every *SA* shares the same master key, which is securely programmed but is not stored in anywhere. The master secret key is mainly used to generate the book key for a segment of eBook content. When a new book is created, *SA* first chooses a random secret and the book key is generated as a hash value of a concatenated string with both the random secret and the *SA*’s master secret, such as *H*(a random secret | *SA*’s master secret). When a user wants to buy the book, the author sends not the book key but only an encrypted random secret. Then, the purchaser should generate the same book key with the given secret and the master secret of the purchaser’s *SA*. This is to authenticate if the purchasing object is a valid service application. In other words, this is to prevent a malicious purchaser from obtaining the original contents using a fake *SA*.

**(2) Why is the book repository necessary?**

We have assumed every registered eBook’s content is uploaded to the book repository, which acts as a content server; the original purpose of the repository is to increase the efficiency of downloading content. Suppose that every author keeps all contents in his or her local storage without the book repository; an author receives every request to buy his or her books, and every buyer should receive the books from the author, and under these circumstances, the overload to the author’s network could be heavy. The book repository acts as a kind of cloud server that can handle many requests and downloading tasks at the same time, and it can be implemented in a decentralized way. The book repository is not a trusted party, that is, it takes no responsibility for the security of stored contents; it has only a control logic, which can validate basic cryptographic protocols such as digital signatures and blockchain hash values. The book repository is necessary for practical use of the proposed system.

**(3) Why does a purchaser need to receive a random book piece from the book repository?**

The proposed purchasing protocol is performed by three parties: a buyer, an author, and the book repository. To validate the book for purchase, the buyer requests a randomly selected book piece, then the book repository responds to the buyer with the corresponding encrypted piece and its hash value: This process is the most crucial part of the proposed system. After the purchase has been completed, the buyer downloads the entire eBook contents from the book repository. In other words, from the buyer’s point of view, it is necessary to verify not only the content for purchase but also the integrity of the contents stored in the book repository; that is, the buyer should be able to verify if the stored contents are the original contents that the author uploaded. Our protocol resolves all of these challenges. First, if the random book piece the book repository gives is restored correctly, that is, if the hash value of the restored content is the same as the given hash value, the buyer can validate the correctness of the given book key as well as the integrity of the content stored in the repository because only the author can create the hash value of each eBook piece; if the restored content is meaningful, then the content is validated. What if not the book repository but the author provided the buyer the random piece during the purchase process? The buyer can validate the given book key for the encrypted book piece given from the author, but, the buyer cannot be sure that the other contents stored in the repository are all valid. What if the buyer receives the encrypted book piece from the author and receives the hash value from the book repository? This is the same as the previous situation: The buyer still cannot be sure of the association of the encrypted content stored in the book repository with the hash. Therefore, obtaining a random piece of eBook content from the repository is necessary.

**(4) What if a user uploads plagiarized content?**

Firstly, a user cannot obtain the original text file of the purchased eBook content without knowing the master secret key of the service application. That is, making a copy of the original text file of the purchased eBook content is impossible. A user, however, can read the purchased book, create new plagiarized eBook content, and publish it as new eBook content. If the plagiarized eBook is exactly same as the existing eBook content, since the hash value of the existing creation is the same as that of the plagiarized creation, it is possible to determine whether plagiarism is performed by comparing the hash values. Plagiarism detection in this way, however, is very inefficient and impractical. Even with a slight change in the text, the hash value is different, so it is not possible to detect plagiarism unless it is a file copy. The proposed system does not automatically detect plagiarized content and does not prevent users from uploading plagiarized content. Further research is needed to solve the plagiarism detection. Typically, user review and reputation systems can be applied to the proposed system.

## Evaluation and analysis

### Security analysis

In this section, we analyze how the proposed eBook transaction system satisfies the suggested security requirements.

#### (1) Ownership, Authentication, and Authorization

First, when a new eBook is created, book information is created including book title and id, author's id and public key, produced, and digitally signed by the author; then the information is broadcast to all users on the network and recorded on the blockchain named as *B_Chain*. Thus, any user can authenticate the owner of each eBook publicly.

Second, any buyer who performed the purchase process correctly obtains a valid purchase transaction and its blockchain containing the transaction block hash. Because the transaction includes signatures generated by both the buyer and the author, a legitimate purchaser can be authenticated by validating the transaction and the blockchain.

Lastly, the only user who possesses both a valid book key for the purchased eBook and a valid contract chain containing the purchase transaction of the book can read the eBook; the book key is required to decrypt the purchased eBook, and the contract chain is necessary to obtain the remainder of the encrypted eBook contents from the book repository. The purchase process consists of two steps: (1) obtaining a book key for an eBook and (2) performing the agreement to the eBook purchase between the buyer and the author. A malicious buyer may perform only the first step and stop the rest of the contract process; then, the contract will not be completed so that a valid contract chain containing the transaction will not be produced. Even though the buyer has received a valid book key, it is not possible to download all the rest of encrypted eBook contents from the book repository without a valid transaction and its blockchain. Therefore, only the legitimate purchaser who has completed the purchase process normally has a right to read the purchased book.

#### (2) Confidentiality of digital content

Basically, every eBook has been encrypted with its book key and stored in the book repository. Every eBook is communicated over the network as encrypted with a receiver’s public key. The book key is randomly generated for each eBook based on the master key of the service application. In addition, the book key is delivered to a buyer as encrypted with the public key of the buyer’s *SA*; thus, only a valid *SA* that knows the *SA* master key can reveal the book key. On top of it, the purchased eBook is re-encrypted for a new key chosen by the buyer’s *SA* and saved in the buyer’s local storage; thus, the purchased eBook can be opened only by the buyer’s *SA*. Also, the new book key is also encrypted with the *SA*’s public key. That is, all information related to the eBook is stored as encrypted so that it is impossible for even a legal buyer to obtain the original unencrypted content without knowing the *SA* master key.

#### (3) Non-forgeability of digital content

As mentioned above, because every eBook is released as encrypted with its own book key, accessing and forging the original content is impossible without knowing the corresponding key. Forging the contents stored in the book repository is also infeasible because eBook uploading requires the corresponding book information digitally signed by the book. Thus, it is impossible to forge already released eBook contents without knowing the private key of the author.

#### (4) Verifiability of digital content

Each eBook is released with the hash value of the original content, and the author digitally signs the book information; with the signature, the buyer can verify the author of the book. During the purchase protocol, the buyer’s *SA* generates the purchased book’s book key and decrypts a randomly selected book piece, allowing the buyer to directly verify if restored content is meaningful. When a buyer has downloaded all encrypted book pieces from the book repository, the buyer can verify the integrity of the downloaded contents by comparing the hash value of the revealed content with the published hash value of the original content.

#### (5) Readability

The purchased eBook is only readable by the buyer’s *SA* because only that *SA* can decrypt the corresponding book key with its private key and present the original content. The purchased contents are stored in the buyer’s local storage, so users can read the books any time.

#### (6) Non-forgeability of transaction

The eBook purchase is an interactive process between a buyer and an author, and the process requires consensus. If and only if both sides generate valid digital signatures about the eBook purchase contract is the contract added to the contract chain. According to the basic nature of the blockchain technology, a valid contract chain can be produced if and only if a valid proof of work about a block containing the contract is generated. Thus, forging a transaction requires forging digital signatures of both sides without knowing the private keys of either side and forging the blockchain as well, whereas this is impossible due to the security of the cryptographic digital signature and of the Blockchain. Therefore, our eBook transaction is non-forgeable.

#### (7) Verifiability of transaction

As mentioned above, the eBook contract contains the digital signatures of both a buyer and an author, and the contract is permanently stored in the contract chain. Thus, the contract can be always verified with the public keys of both sides and current contract chain due to the nature of the blockchain.

#### (8) Forbidden of illegal distribution

Ultimately, users cannot obtain the original contents of eBooks that they did not create from their local storages because users cannot obtain valid book keys of the purchased eBooks. Therefore, it is impossible to redistribute the original contents of purchased eBooks. In other cases, the encrypted contents stored in the user’s local storage may be redistributed, but the book key cannot be restored because the key is encrypted with the private key of the user’s *SA*. The encrypted content can be decrypted only by the *SA* of the original purchaser, and the redistribution is meaningless because other *SA*s cannot decrypt the content normally.

### Simulated performance analysis

In this section, we examine the simulated performance of the proposed eBook transaction system. Our model should be serviced in real-time, so we analyze the average execution time for the following seven main actions: eBook generation, eBook registration transaction (*BTRS*) broadcasting, eBook upload to the book repository, eBook purchase transaction (*CTRS*) generation, blockchain update, eBook download from the book repository, eBook resave. Five experimenters randomly repeated the eBook registering and purchasing process. The experimenters performed the registration and purchase process at least 10 times for each eBook size. Our experimental results show the average value of all execution times performed by the experimenters for each main action. In particular, the eBook upload and the eBook download require additionally transaction verification with blockchain. In other words, *BTRS* verification using *B_Chain* and *CTRS* verification using *C_Chain* are required before the eBook upload and the eBook download, respectively. Thus, each transaction verification time will be analyzed separately. We describe first the experimental environment including user information and eBook information, and analyze our experimental results in detail. The detailed experimental environment for the simulation is described in Table 4.

**Table 4:** Simulation environment parameters.

****Parameter****	****Values****
****OS****	Windows 10
****RAM****	16 GB
****CPU****	Intel i7-6700
****The number of nodes****	5
****Development tool****	Java
****Public key encryption / decryption****	ECIES
****Digital signature****	ECDSA
****Symmetric key encryption / decryption****	AES 256
****Message hash algorithm****	SHA 256
****User information type****	JSON format
****The original eBook type****	Text file (.txt)
****eBook information type****	JSON format
****eBook file size****	50KB, 100KB, 500KB, 1MB, 3MB, 5MB, 10MB
****The level of difficulty****	3, 4, 5
****Blockchain and digital coin****	Self-developed coin and blockchain mechanism

Our system begins with the installation of the proposed *SA*, which registers a new user, creates new user information, and broadcasts it to existing users. The *SA* obtains a current user list from a predefined genesis server; the user information contains user’s id (*uid*), user’s public key (*upk*), user’s network ip and port (*ip* and *port*), and the public key of the user’s SA (*spk*). The user information is produced as a JSON format as shown in Table 5 and is broadcast to all existing users. Then, users update their current user lists.

**Table 5:** An example of user information in JSON format.

{"uid":"soyoung", "upk":"MEkwEwYHKoZIzj0CAQYIKoZIzj0DAQEDMgAExxTLAUMcO1NbcXvXY tga0UxOQ3kf18B9J8h65OirAOTz5Cr3xpj00Bw23QMS0jk6", "port":4000, "ip":"000.000.000.000", "spk":"MEkwEwYHKoZIzj0CAQYIKoZIzj0DAQEDMgAE9Iayc7zj+q5tv8OxV9WcB LdAWAwUC0ZOxsbxMKojti9UOin8DkIJB0r0AOkYgCTJ"}

Whenever a user creates new eBook content, the book registration transaction containing the book information is broadcast to all users on the network, and encrypted eBook pieces are uploaded to the book repository. The book information shown in Table 6 contains book id (*bid*), book title (*title*), author’s id (*uid*), author’s public key (*upk*), book price in coins (*price*), signature for encrypted book pieces and hash list (*signatureStr*), book summary (*summary*), book size in bytes (*size*), the total number of book pieces (*numOfPices*), and timestamp. Each encrypted book piece is filed in bytes, and its file name consists of its *bid* and its piece number.

**Table 6:** An example of eBook Information in JSON format.

{"bid":"5d84cd965061bb352e88858463dac17642836fba408f2881f67977f1ea03762a", "title":"p_50_2", "uid":"Alice", "upk":"MEkwEwYHKoZIzj0CAQYIKoZIzj0DAQEDMgAEFDJG1IAURDXX2460oHP8kjc2RgE1yviprY3x4cr/GE52kfBxkwhmVi1nXYVXRnC/", "price":2.0, "signatureStr": "MDQCGFiiTGH1bOXfmc182WqcOzMMliWWI756hQIYUNarqqI65zQ/wdABhPpsNZgoeFnDpdzM", "summary":"This is a test book file.", "size":51253, "numOfPieces":103, "timestamp":"2019-11-24 13:55:37.213"}

An eBook is divided into small book pieces, and the number of book pieces is determined according to the book size as shown in Table 7.

**Table 7:** The number of file pieces.

	****50KB****	****100KB****	****500KB****	****1MB****	****3MB****	****5MB****	****10MB****
****The number of pieces****	103	103	257	513	615	1025	2050

The main processes of our system are (1) eBook registration, (2) eBook purchase, and (3) eBook download and storage. Registering an eBook consists of generating a new eBook, updating *B_Chain* with a new book registration transaction, and uploading the eBook to the book repository. The eBook purchase is divided into two steps: a new eBook purchase transaction and *C_Chain* update to include the new transaction. The first step entails validating the eBook and the mutual transaction between a buyer and an author; it involves the buyer reading and confirming the revealed content. If the first step is performed correctly, the second step takes place: *C_Chain* is updated with the addition of a new block that contains the transaction. In our simulation, the buyer finally completes and sends the eBook purchase transaction to the author, and then the author creates and replies with a new block by mining the proof-of-work of the block for practicality and efficiency. As soon as the eBook purchase is complete, the eBook download is performed automatically. More specifically, the execution time of each detailed step is determined as follows:

- **eBook generation time** = load contents from text file + generate *bid* + generate book key + generate encrypted book pieces + generate book registration transaction (*BTRS*)
- ***BTRS* broadcasting time** = send book information JSON file to a single user
- ***B_Chain* and *C_Chain* update time** = create block + mine block
- **eBook upload time** = generated book upload request + send request and list of encrypted book pieces + verify request with *B_Chain* + save book pieces in files
- **eBook purchase transaction (*CTRS*) generation time** = generate book purchase request + generate and verify signature + send request + encrypt book key + generate contract message + generate and verify signature + decrypt book secret and generate book key + decrypt random book piece + confirm the decrypted book piece + generate *CTRS*
- **eBook download time** = generate book download request + send book request + verify request with *C_Chain* + download whole pieces of eBook
- **eBook resave time** = decrypt eBook + generate new book key + encrypt the book with new book key + save the encrypted book and book key in local storage

**Fig 9:**
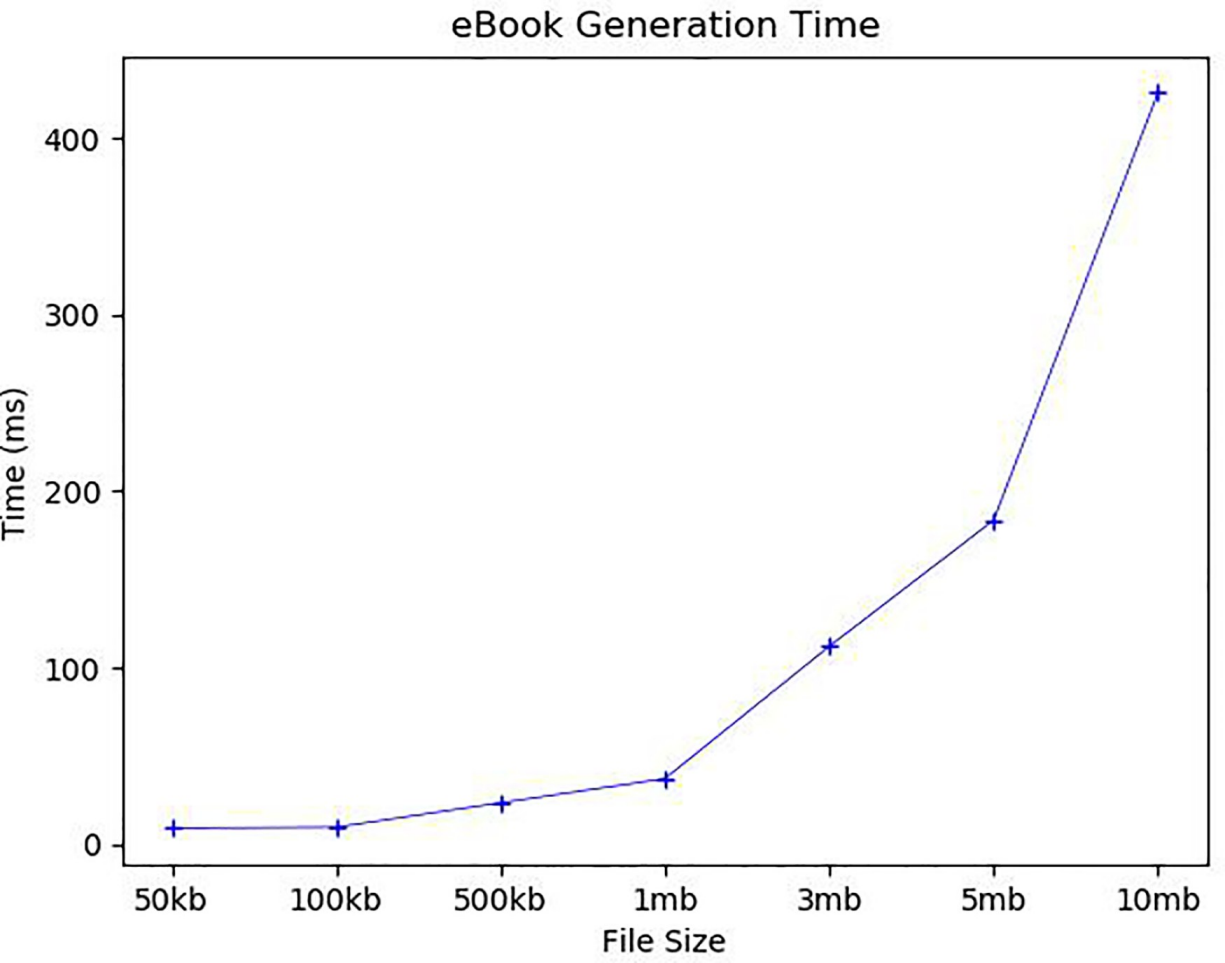
eBook generation time.

**Fig 10:**
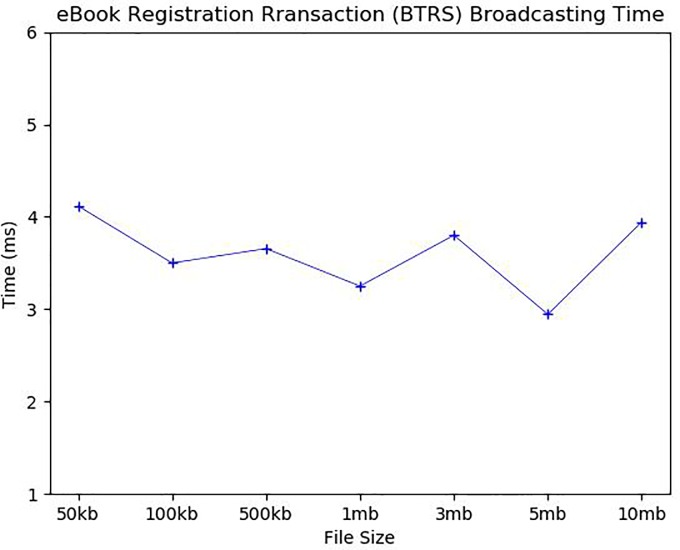
eBook registration transaction (*BTRS*) broadcasting time.

We now analyze the average execution time of each detailed step. Fig 9 first shows the average time to generate an eBook: It took 9.1ms and 9.7ms to generate 50 and 100 kb eBooks, 23.5ms for eBooks of 500 kb, 37.1ms for 1 mb, 112.1ms for 3 mb, 183.6ms for 5 mb, and 425.6ms for 10 mb. Since the most time-consuming task in the eBook generation is to create encrypted book pieces, the eBook generation time increases proportionally to the number of book pieces and the size of each book piece. The average *BTRS* broadcasting time to a single user is about 3.5ms as shown in Fig 10. Because *BTRS* contains only the book information regardless of eBook size, the broadcasting time is very dependent on the network environment. We conducted our simulation on the same local network, so the broadcasting time was very short. *B_Chain* for broadcasted *BTRSs* is updated periodically. The *B_Chain* update time will be analyzed along with *C_Chain* update time later.

**Fig 11:**
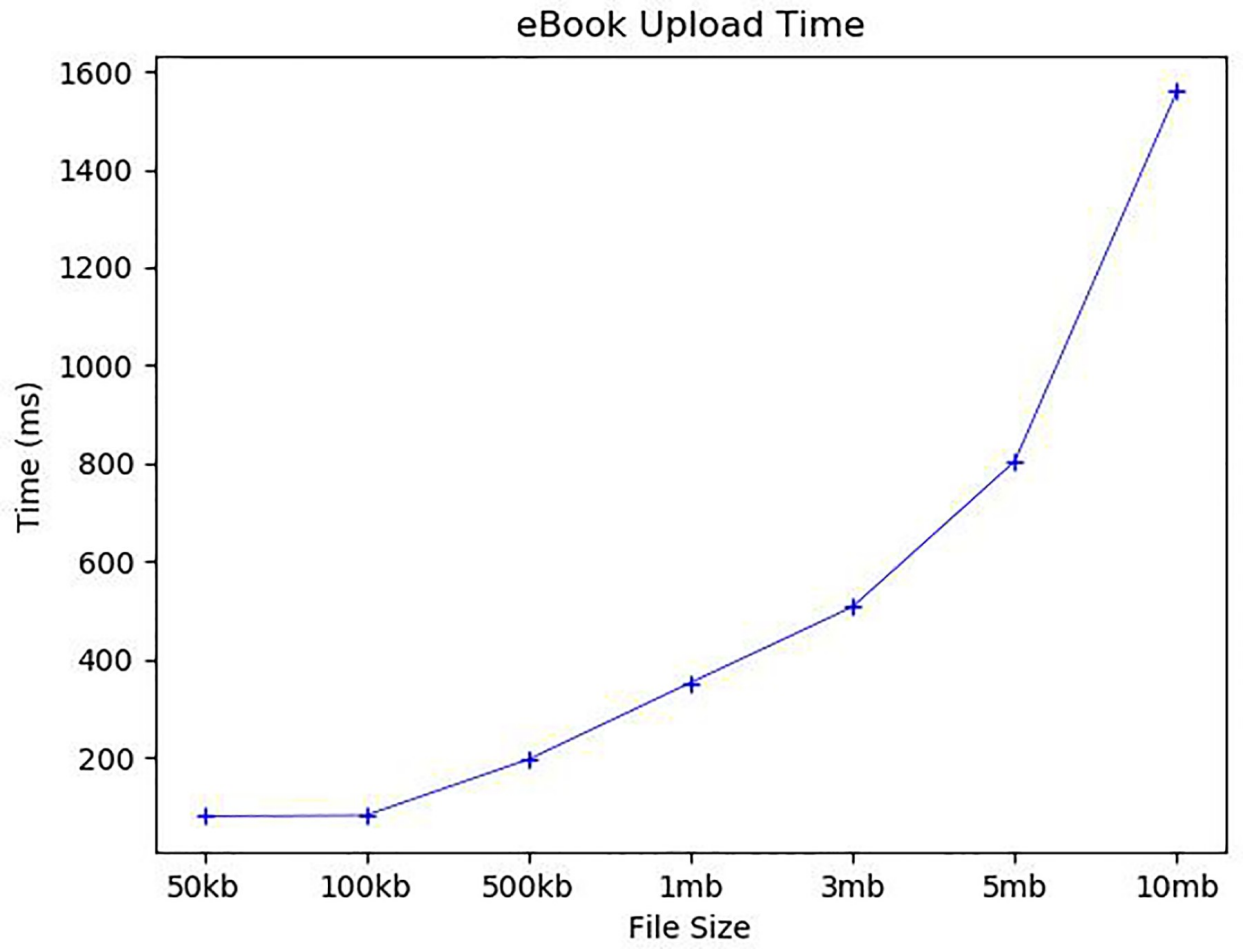
eBook upload time.

**Fig 12:**
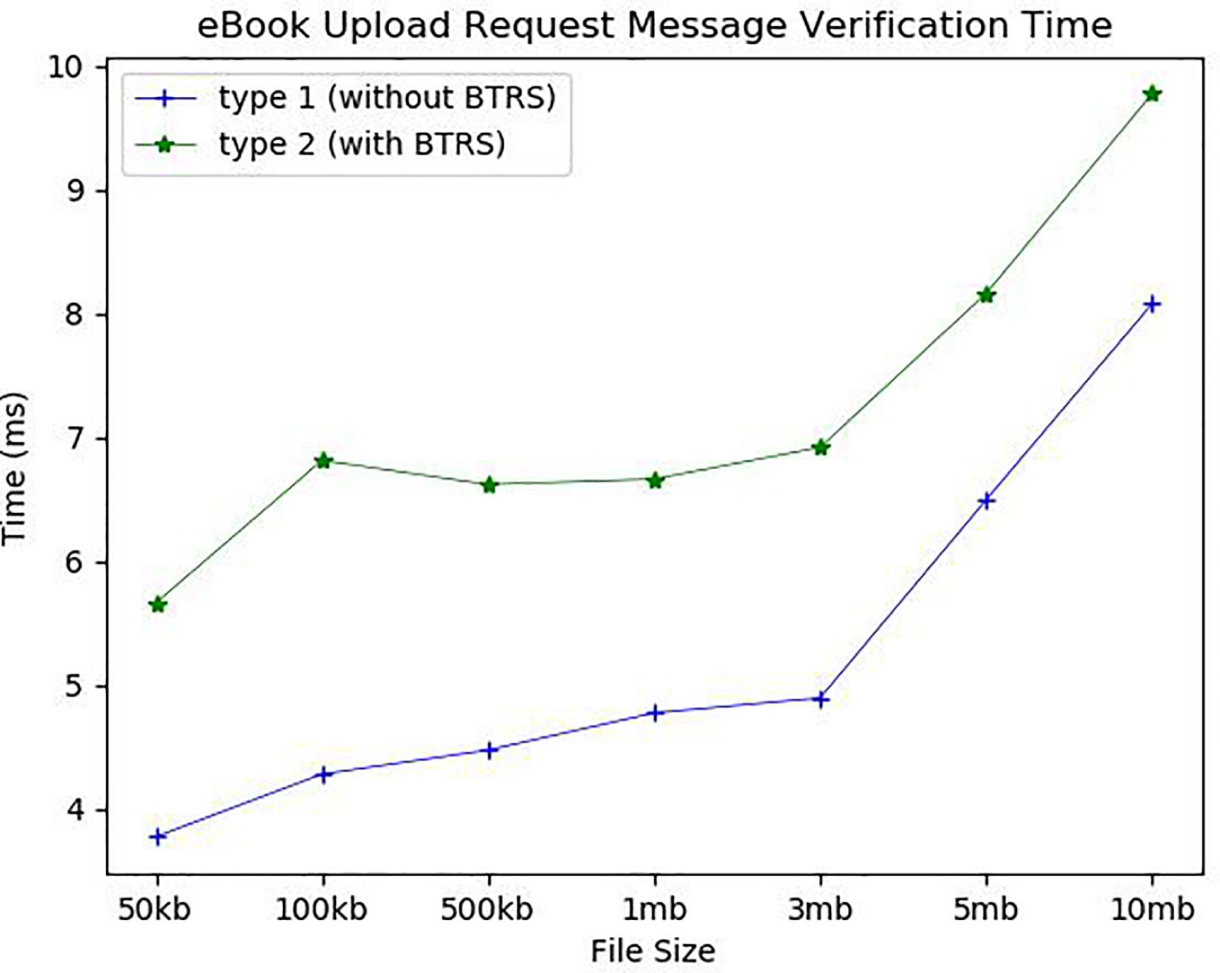
eBook upload request message verification time.

Fig 11 shows the average time to upload the eBook to the book repository. The encrypted eBook file pieces and the hash list of the file pieces are delivered to the book repository as encrypted with the book repository’s public key. The length of hash list increases in proportional to the number of file pieces. The eBook upload includes verifying the upload request message along with uploading the encrypted file pieces. The upload request verification consists of decrypting the delivered message and validating the upload request message with *B_Chain*. If the verification is valid, each encrypted file piece is saved as a separate file in the book repository. Fig 12 shows the average eBook upload request verification time for two types: Type 1 (without *BTRS*) assumes for the book repository to store all *BTRS*s recorded in *B_Chain* but Type 2 (with *BTRS*) assumes to keep only the hash of every *BTRS*. Thus, for type 2, *BTRS* of the uploaded eBook should be delivered to the book repository and additional verification for the given *BTRS* itself is necessary. For Type 1, it took around 3.7ms for 50 kb, 4.3ms for 100 kb, 4.5ms for 500 kb, 4.7ms for 1mb, 4.9ms for 3mb, 6.6ms for 5mb and 8ms for 10 mb. For Type 2, it took around 5.6ms for 50 kb, 6.8ms for 100 kb, 6.6ms for 500 kb and 1mb, 6.9ms for 3 mb, 8.1ms for 5 mb and 9.7ms for 10 mb. The computational overhead for Type 2 verification is around 3ms. For the eBook upload, it took about 81.25ms and 83ms to upload 50 and 100 kb, 196.8ms for 500 kb, 353.25ms for 1 mb, 508.2ms for 3 mb, 804.5ms for 5 mb, and 1558.75ms for 10 mb. The eBook request verification time and eBook upload time for 5 mb and 10 mb files increased more significantly than others because the number of file pieces and eBook size nearly doubled than 3 mb and 5 mb eBooks, respectively. In addition, the longer hash list was added to those files. Our experimental results show that the eBook upload time is mainly affected by the number of file pieces and the size of each file piece. We will continue further research to find the optimal number of file pieces that ensure both the eBook safety and communication efficiency.

**Fig 13:**
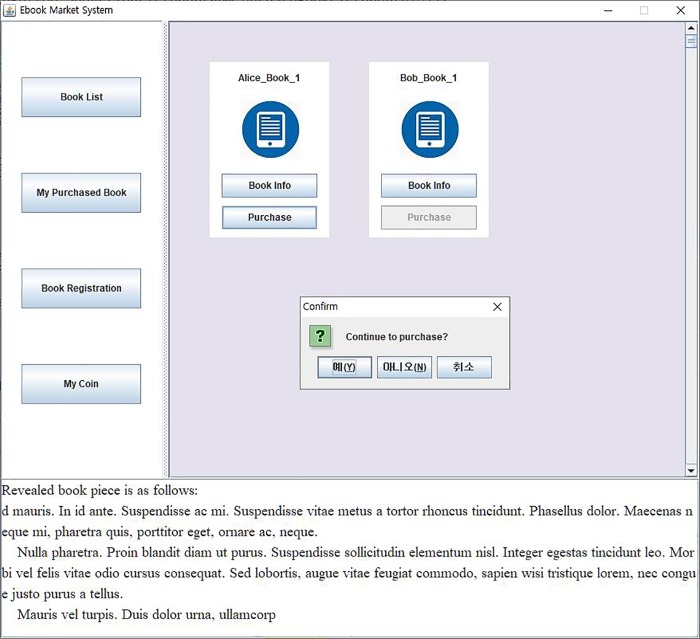
User confirmation of the decrypted book piece.

**Fig 14:**
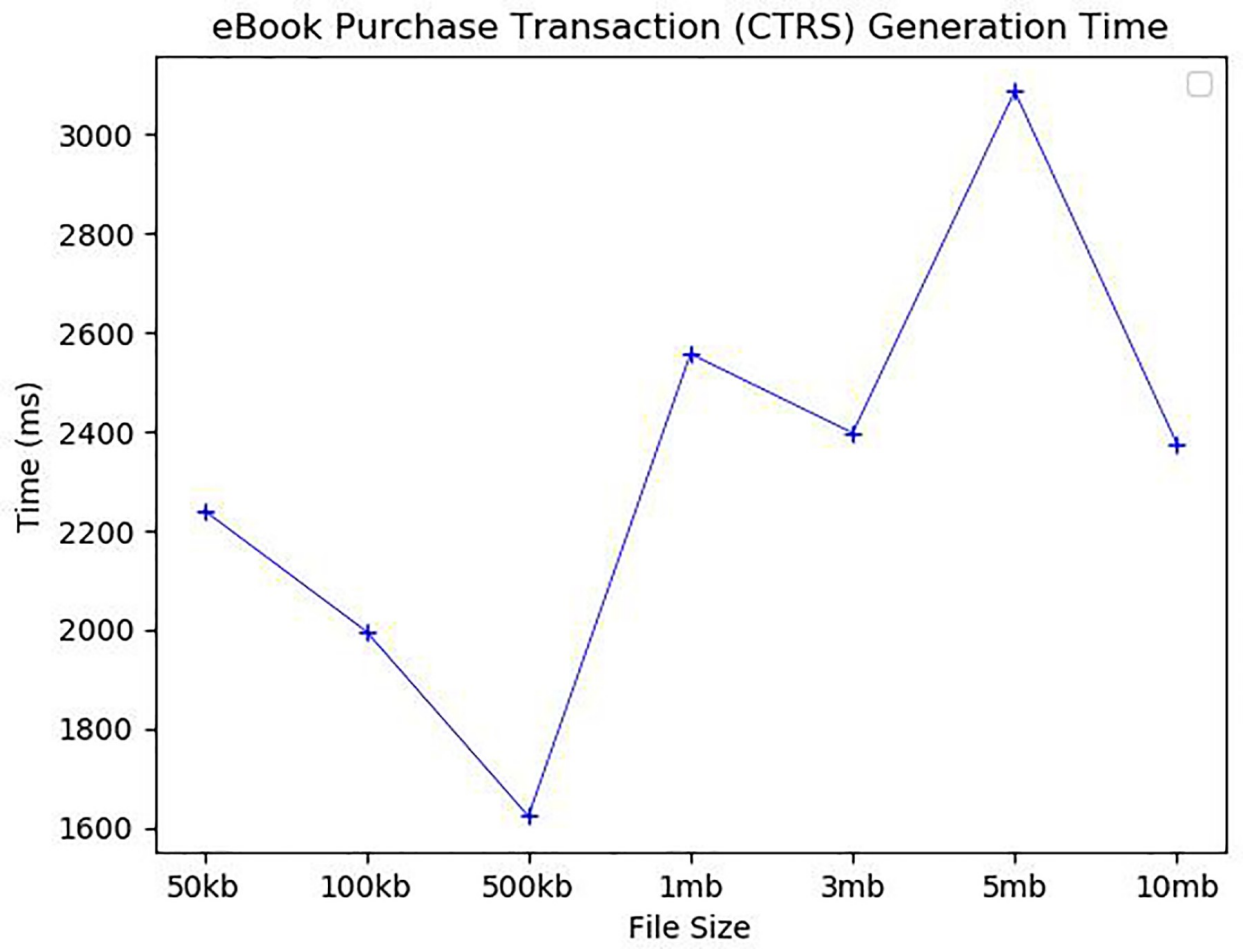
eBook purchase transaction (*CTRS*) generation time.

**Fig 15:**
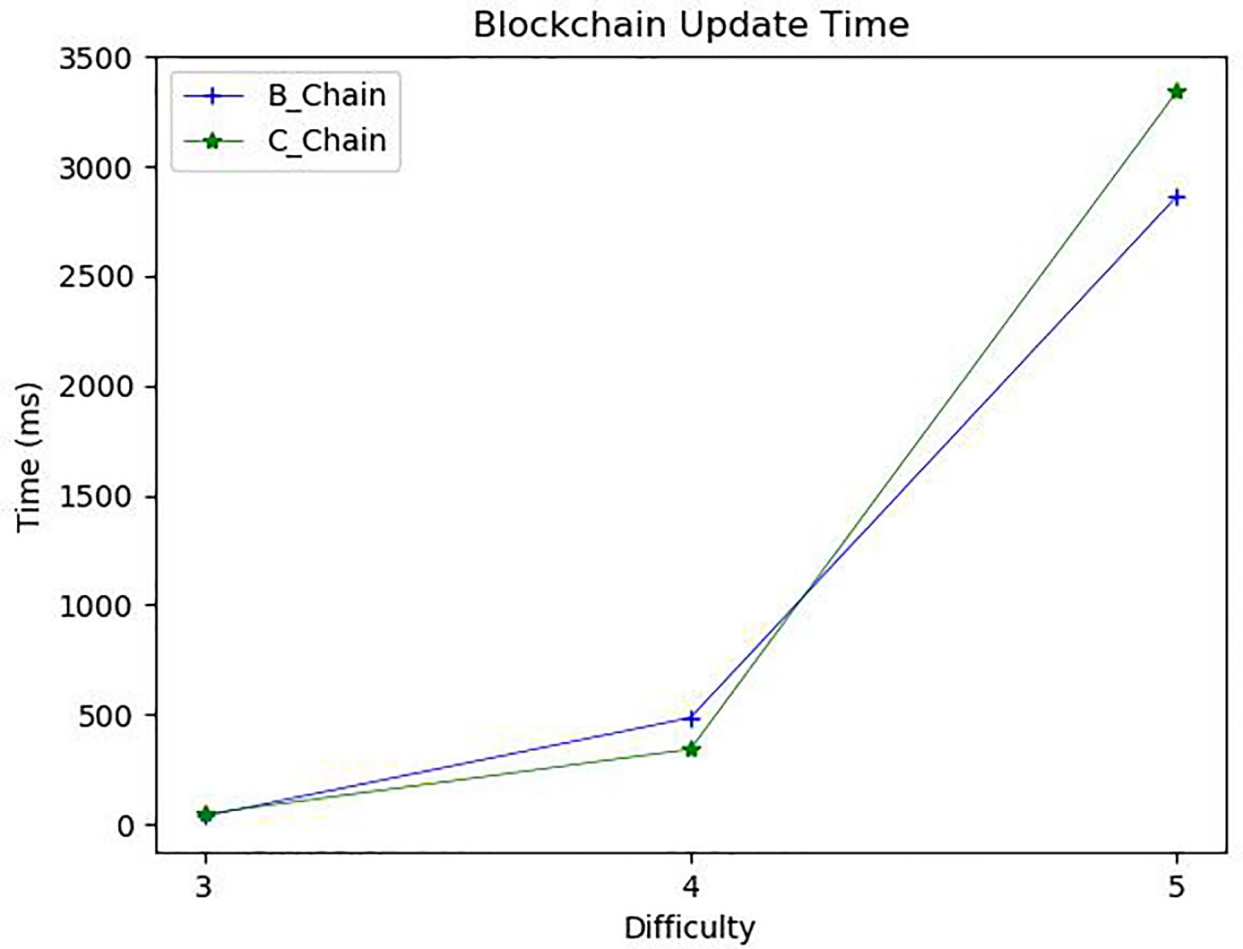
Blockchain update time.

As mentioned above, eBook purchase consists of generating an eBook transaction and updating a corresponding blockchain. The most time-consuming task in generating the transaction is the buyer’s content verification, during which the buyer reads partially revealed contents, verify if the content makes sense, and then confirms purchase by clicking “yes” in the confirmation dialog window. Fig 13 shows the confirmation performed by *SA*. The revealed content is presented at the bottom text filed of *SA*. Thus, the transaction generation time depends heavily on the buyer’s response behavior. In our experiments, the experimental participants usually responded in about 2 seconds as shown in Fig 14. Once the buyer and the author have made an agreement on their transaction, a valid transaction is completed, and then, the blockchain is updated. A new block is generated that contains the new transaction, and that block is then added to the existing blockchain. In the blockchain update, the mining block, which finds a random nonce satisfying a predefined difficulty, is the most time-consuming. We analyzed the blockchain update time according to levels of difficulty 3, 4, and 5. The block mining time was very jagged, but the average execution time was different depending on the level of difficulty as shown in Fig 15. For *B_Chain* update, it took about 37ms for level 3 of difficulty, 485ms for level 4, and 2862ms for level 5. Similarly, for *C_Chain*, it took about 47ms for level 3, 342ms for level 4, and 3340ms for level 5.

**Fig 16:**
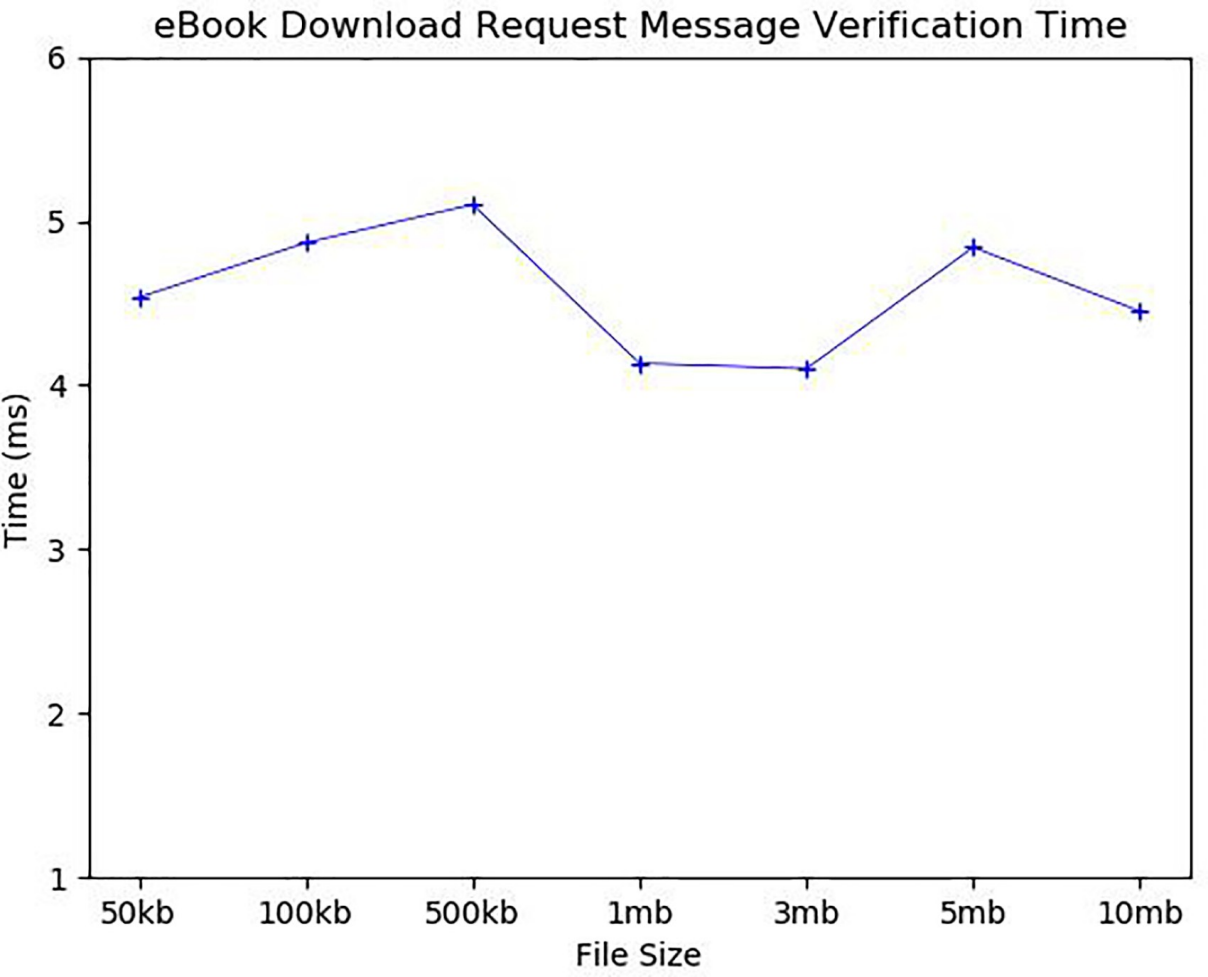
eBook download request message verification time.

**Fig 17:**
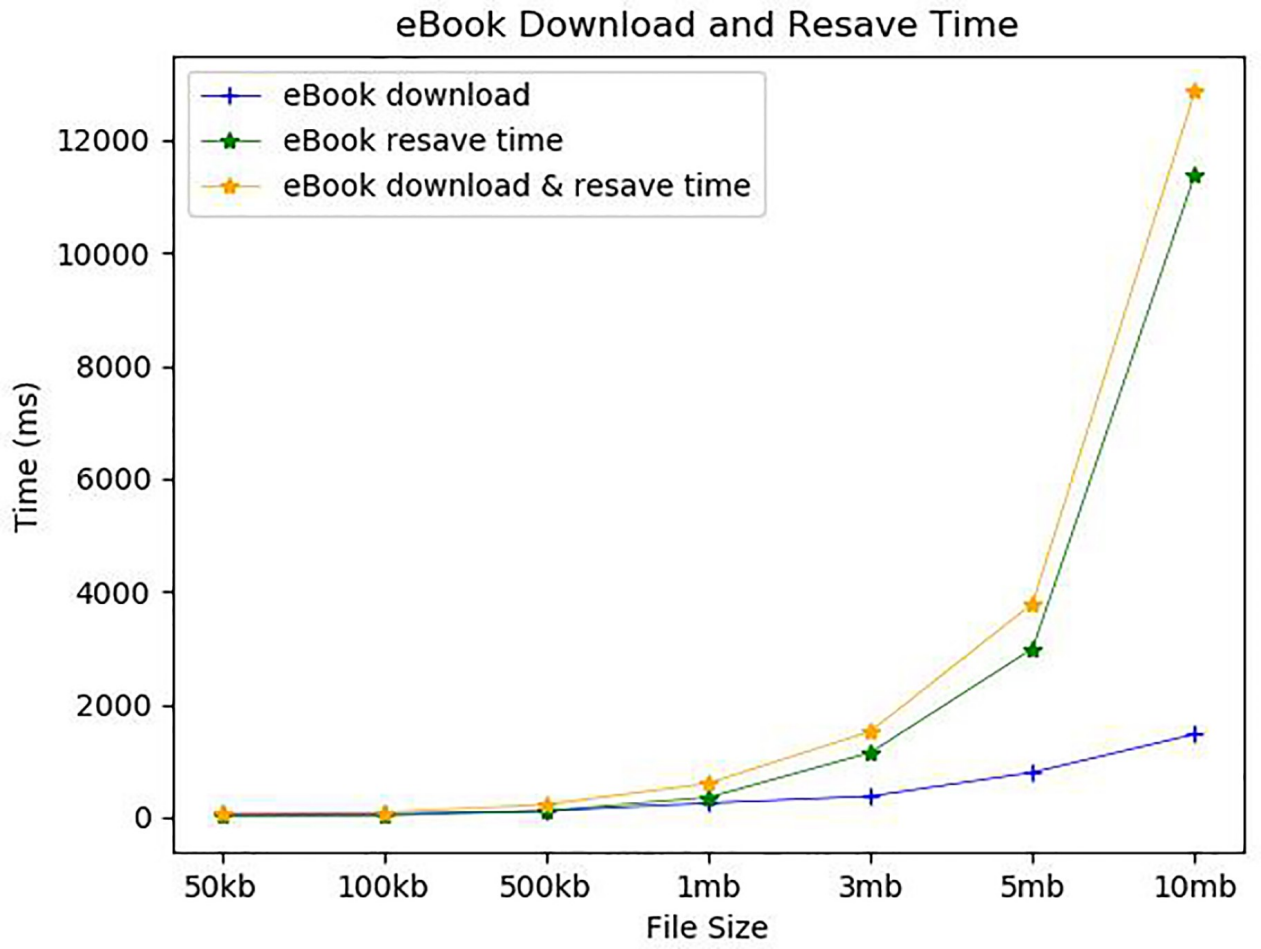
eBook download and resave time.

Fig 16 and Fig 17 show the average eBook download request message verification time and eBook download and resave time according to various eBook sizes. The eBook download also requires the verification of the eBook download request. We assumed that the book repository only keeps the hash values of all *CTRS*s recorded in *C_Chain*. Thus, the eBook download request message contains the corresponding *CTRS*. As shown in Fig 16, the *CTRS* validation took about 4.5ms regardless of eBook size. On the other hand, eBook download time is mainly affected by the number of eBook pieces and the size of each file piece, so it increases significantly with the number of eBook pieces. It took about 57ms and 64ms for 50 kb and 100 kb eBooks, respectively, but it took 110ms for 500 kb, 250ms for 1 mb, 374ms for 3 mb, 797ms for 5 mb, and 1473ms for 10 mb. The downloads took less time than the uploads because the download only requires communication and not include the file storage step. The downloaded encrypted book pieces are re-encrypted with a randomly chosen new book key and saved in the user’s local storage as encrypted. More precisely, each book piece must be decrypted and then combined into whole content, a new book key should be generated, the whole content must be encrypted again with the new book key, and finally, both the re-encrypted book content and its book key are saved in files. Thus, this task increases in proportion to the number of file pieces and the size of each file piece, and in fact, it took about 20ms for a 50 kb eBook, 27ms for 100 kb, 114ms for 500 kb, 352ms for 1 mb, 1145ms for 3 mb, 2990ms for 5 mb, and 11386ms for 10 mb. Our simulated results show that every action related to direct eBook transaction can be accomplished in a reasonable time.

Finally, we compare the features of the proposed model with Publica [37], which is the most similar blockchain-based peer-to-peer eBook publishing product. Table 8 summarizes the features of both models.

**Table 8:** Summary of the features of the proposed model and Publica.

****Features****	****Publica [37]****	****The proposed model****
****Trustee****	Publica	X
****Self-publishing****	O	O
****Direct payment to author****	O	O
****Payment****	Digital coin	Digital coin
****Copyright management****	Publica, Publisher or Author	Blockchain
****Purchase contract management****	Blockchain	Blockchain
****Right to read****	Book token	Book key
****Book key verification during purchase****	X	O
****Content verification during purchase****	X	O
****Content re-encryption****	unknown	O

The proposed model is meaningful in that it protects the copyright of paid contents as well as the safety of direct payment based on blockchain technology in trustless environment, and provides the function that buyers can directly verify the validity of paid contents and the corresponding book keys during purchasing.

## Conclusions

We have here introduced a blockchain-based eBook transaction system that enables direct eBook purchase between authors and readers without trusted parties. In the proposed system, any user can be either an author or an eBook content buyer. Because of the direct transaction, no intermediate costs occur, but the security of eBook contents and eBook purchase transactions cannot be protected. Thus, we proposed a secure and reliable eBook direct transaction system that overcomes all security vulnerabilities in the absence of trust parties.

The proposed system guarantees the confidentiality of eBook contents; only a legitimate purchaser can read a purchased book. Because the buyer can verify the validity of the eBook content before the purchasing process is completed, authors are also prevented from publishing garbage content. Forging and repudiating previously performed transactions are prevented by the blockchain technology, making eBook purchase transactions securely protected. Because every purchased eBook’s content is newly encrypted and stored by the buyer’s service application, resale or redistribution of the purchased content is systemically prevented. As such, the proposed system secures not only the contents of eBooks but also the rights of buyers and authors.

We examined the security of the proposed protocols and the average execution time for main actions including eBook registration, purchase, downloading, and storage, according to various sizes of eBook contents and different levels of difficulty in blockchain generation. Our implemented results show that all the main operations could be accomplished in a reasonable time.

Currently, only eBook purchases are allowed, but continuous research will be conducted to expand the system to allow eBook rental for a limited time. In addition, studies for evaluating the reputation of eBooks and authors will continue as well.
